# Neighborhood deadwood and yard rewilding modulate commensal microbiomes and inflammatory signals among urbanites

**DOI:** 10.1186/s40168-026-02413-w

**Published:** 2026-05-15

**Authors:** Marja I. Roslund, Laura Uimonen, Laura Kummola, Damiano Cerrone, Ann Ojala, Anna Luukkonen, Ella Holopainen, Aku Korhonen, Reijo Penttilä, Mika Saarenpää, Martti Venäläinen, Hanna Haveri, Juho Rajaniemi, Olli H. Laitinen, Aki Sinkkonen, the BIWE research group

**Affiliations:** 1 Natural Resources Institute Finland (Luke), Latokartanonkaari 9, 00790 Helsinki, Finland; 2 Faculty of Built Environment, Tampere University, Korkeakoulunkatu 5, Tampere, Finland; 3 Faculty of Medicine and Health Technology, Tampere University, Arvo Ylpön Katu 34, 33520 Tampere, Finland; 4 Faculty of Biological and Environmental Sciences, University of Helsinki, Viikinkaari 1, 00790 Helsinki, Finland; 5 Wellbeing Services County of Päijät-Häme, Keskussairaalankatu 7, 15850 Lahti, Finland; 6 Faculty of Medicine, Helsinki University, Haartmaninkatu 4, Helsinki, Finland; 7 Benefa Oy, Tampere, Finland; 8 University of Eastern Finland, Joensuu, Kuopio Finland

## Abstract

**Background:**

Urbanization and biodiversity loss reduce human exposure to diverse microbiomes. Current evidence suggests that the vanishing microbiomes in industrialized populations are a central factor in the rising prevalence of non-communicable immune-mediated diseases. Rewilding has been proposed as an approach to diversify urban microbial communities and promote immunological resilience.

**Results:**

We rewilded 21 urban private yards with deadwood, vegetation, and microbially rich soil. Control yards (15) were analyzed for comparison. We analyzed skin bacteria and oral microbiomes and used vegetation and deadwood inventories, satellite data, and questionnaires to determine the effects of rewilding, living environment, and lifestyle factors on skin and oral microbiota, functional gene pathways, and cytokine levels (IL-6, IL-10). Neighborhood deadwood within 200-m radii around home yards was used as an indicator of environmental biodiversity. Samples were collected before the rewilding in summer and three months later in autumn.

Skin microbial diversity stayed constant and was associated with plant richness in the rewilding group, despite the normal seasonal decline and reduced outdoor time in autumn. Rewilding was associated with a decrease in tetrahydrofolate biosynthesis and salvage, and L-histidine degradation gene pathways and other changes in oral microbiota. In the rewilding group in autumn, picking of berries and fruits was directly associated with immunoregulatory IL-10 and pleiotropic IL-6 in saliva, and neighborhood deadwood abundance with the fatty acid biosynthesis superpathway in oral microbiomes. When groups were analyzed jointly, the diversity of oral microbial functional gene pathways was negatively correlated with IL-6 levels, and neighborhood deadwood abundance was directly linked to skin Gammaproteobacterial taxa, and typically soil-derived *Cytobacillus* sp. CY-G and *Streptomyces* sp. HSG2 in saliva.

**Conclusions:**

Our findings are consistent with the biodiversity hypothesis, suggesting that biodiversity exposure may influence commensal microbiomes and biological pathways involved in host–microbe interactions. Our results suggest that the amount of decaying deadwood in the neighborhood, in addition to conventional measures of greenness and vegetation diversity, may provide advantageous information in studies examining human–environment microbiome interactions. This may inform biodiversity-related ecosystem services related to impacts on human health.

Our findings provide an incentive for future studies and strategic investments for rewilding urban microbiomes to support planetary health.

Video Abstract

**Supplementary Information:**

The online version contains supplementary material available at 10.1186/s40168-026-02413-w.

## Introduction

According to current understanding, biodiversity loss and the lack of microbial exposure in urban ecosystems are core drivers of human microbiome depletion and the epidemic of immune-mediated diseases among urban populations [1–4]. Hence, there is an unmet need to enrich urban environmental microbiota [5–7]. A recent study demonstrated that oral microbiomes reflect a gradient of lifestyles ranging from traditional to industrialized [3]. However, the causal link between living environment and oral microbiome, as well as experimental evidence on how microbially focused rewilding of urban home yards influences oral microbiome composition, its functions, and associated salivary inflammatory signals, remains unexplored. While diet and behavior are likely primary drivers of gut microbiome composition [8], the potential contribution of the external living environment to oral microbiome assembly remains less well understood. Urban rewilding has many benefits both for the environment and humans. These benefits include increased resilience to environmental change and opportunities for urban dwellers to reconnect with nature [8]. Urban, private yards contribute remarkably to local ecology, biodiversity and wellbeing of the residents [9, 10]. Microbially diverse private yards are hypothesized to provide ecosystem services, such as biodiversity support and pathogen suppression, that alleviate microbial deprivation and proinflammatory responses among residents [1, 7, 11]; however, this has remained untested.

Environmental microbial and plant community diversity influence skin and oral microbiota [2, 12–20]. Recent studies demonstrated how rewilding of urban daycare yards diversifies children’s skin microbiota and enhances immune regulation [13, 14]. The core reason for the enhanced immune regulation seems to be the exposure to diverse microbiome per se; when study subjects were exposed to microbially rich sandbox sand [20] or growing media with high microbial diversity [16], immune regulation was enhanced only in the intervention groups, not in the placebo groups.

Studies exploring the human immune system have frequently used plant diversity indices or plant species richness as an indicator of exposure to diverse microbiomes [2, 21–24]. Since a recent review summarized the results of such studies as conflicting [22], differences in vegetation diversity hardly provide a complete explanation for the difference in services provided by urban and rural ecosystems. Since the accumulation of decaying plant debris is recognized as an important driver of biodiversity, the importance of decomposing plant matter could provide an alternative explanation why urban ecosystems seldom balance human microbiomes and immune modulation [7]. Indeed, decaying wood is recognized to provide habitats for numerous fungi, saproxylic invertebrates, and prokaryotes, particularly bacteria, and thus represents a key reservoir of environmental microbial diversity [25, 26]. In addition, wood-inhabiting polypore fungi represent a group of basidiomycetes with easily observable poroid basidiocarps developing on decaying wood, and they have often been used as biodiversity indicators of forested habitats in the Nordic region [27]. Comparative studies have shown that people living in environments hosting rich microbiomes, such as agricultural or more biodiverse landscapes, have a lower probability of certain immune-mediated diseases [2, 28]. While these environments are not defined by deadwood per se, together these findings suggest that increasing microbially rich habitat features within urban settings could enhance human microbial exposure. Accordingly, the evaluation of ecosystem services provided by living environments should extend beyond plant species richness to include indicators of decaying plant matter, e.g., deadwood and wood-inhabiting polypore fungi [27, 29].

In parallel with proper measures of biodiversity, measures of human microbiome and immune function must be feasible and reflect environmental exposure to potential modifiers. Skin and oral microbiomes are directly and continuously exposed to the surrounding environment and are therefore sensitive to changes along urbanization and biodiversity gradients [3, 14, 18, 20]. The skin serves as a primary interface between the body and the outdoor environment, where contact with vegetation, soil, and airborne particles can rapidly influence its microbial community composition [12, 30]. Similarly, the oral cavity integrates environmental, dietary, and behavioral exposures and represents a key entry point to the gastrointestinal and respiratory tracts [3, 31]. Previous biodiversity interventions at daycare centres have been shown to modify the skin and oral microbial communities of children [14]. However, no studies have yet assessed whether home yard rewilding can shape adult skin and oral microbiomes, including both taxonomic composition and functional gene pathways.

Saliva inflammatory markers provide a noninvasive alternative to blood cytokines for assessing inflammatory responses [32–34]. Salivary cytokine levels are responsive to environmental and microbial exposures and have been associated with local and systemic inflammatory processes, mucosal immunity, and neuroimmune signaling [32–34]. Thus, in the context of rewilding interventions, skin and oral microbiomes together with salivary cytokines offer biologically relevant biomarkers of host microbial and immune responses [32–34].

The impact of microbially focused rewilding of private yards, including changes in garden management practices, on health-associated commensal microbiomes and salivary cytokine levels has never been tested in an intervention trial. We rewilded urban private yards with diverse vegetation and decaying deadwood and plant residues. We used satellite data and vegetation, deadwood, and polypore inventories to determine the living environment of the participants and biodiversity around home yards. Questionnaires were used to evaluate the yard management practices and lifestyle of yard owners. Based on previous comparative studies [2, 15, 32, 33] and intervention trials [13, 14, 16, 20], we hypothesized that yard rewilding increases the diversity of health-associated skin microbiota, particularly that alpha- and gammaproteobacterial diversity would be higher in the rewilding group than in the control group. We further hypothesized that the rewilding is associated with higher salivary cytokine levels and increased relative abundance of microbial taxa and functional gene pathways related to immune function, resulting in different temporal changes between the rewilding and control groups. We also hypothesized that plant species richness and the abundance of neighborhood deadwood are indicators of microbial exposure and thus associated with commensal microbiome profiles.

## Results

Thirty-six healthy volunteers aged between 33 and 70 participated in the study (Table 1). Participants were assigned either to an intervention group, hereafter referred to as the rewilding group, or to a control group. The majority of land cover around all yards for all radii measured (100–2500 m) was artificial surfaces, particularly discontinuous urban fabric (Table 1). Yard characteristics were similar between treatment groups at baseline, except that the control group had approximately 16% more manicured yard area (*p* = 0.02, padj = 0.05; Table S1). According to the questionnaires, gardening was practiced as often, i.e., at least weekly, in both groups during the study period. The study groups did not differ in the amount of neighbourhood deadwood or in polypore species richness within a 200 m radius around the yards (excluding the private home yards themselves) during the study period (*p* > 0.1). At baseline, yard management practices, activities in the yard, nature visits, and animal and soil contacts were similar between treatment groups (*p* > 0.05).

**Table 1:** Characteristics of the study participants**.** Age and land cover categories around home yards with radius 500 m are presented as mean ± standard deviation. The land cover categories surrounding the private yards were analyzed with the preclassified Coordination of Information on the Environment (CORINE)

	**Rewilding**	**Control**	**Total**
Gender, male	5	4	9
Gender, female	16	11	27
Gender, non-binary	0	0	0
Age	46 ± 11	52 ± 13	48 ± 12
Pet ownership	12	3	15
Land cover categories 500-m radius:			
City:			
- Helsinki	8	5	13
- Lahti	11	8	19
- Turku	2	2	4
Artificial surfaces%:	94 ± 16	95 ± 7	95 ± 13
- Discountinous urban fabric%	59 ± 21	70 ± 16	64 ± 20
- Industrial or commercial units	26 ± 20	20 ± 21	24 ± 20
- Green urban areas%	9 ± 16	3 ± 10	6 ± 14
Forests%	2 ± 8	1 ± 5	2 ± 6
Agricultural areas%	3 ± 13	1 ± 4	3 ± 10

By the end of the study period (90 days post-intervention; autumn), the rewilding group had planted more often compared to the control group (GLM: *p* = 0.002, padj = 0.05, *R*^2^ = 0.22; Table S2). Based on participant questionnaire responses, none of the participants used pesticides. Insecticides were used rarely or never. No inorganic fertilizers were reported to be used in the rewilding group during the study period, whereas participants in the control group reported using inorganic fertilizers no more than three times in a month. Time spent at the home yards decreased by the end of the study period in both the rewilding and control groups (Table S2).

### Neighborhood deadwood and land cover were associated with commensal microbiota

**Fig. 1:**
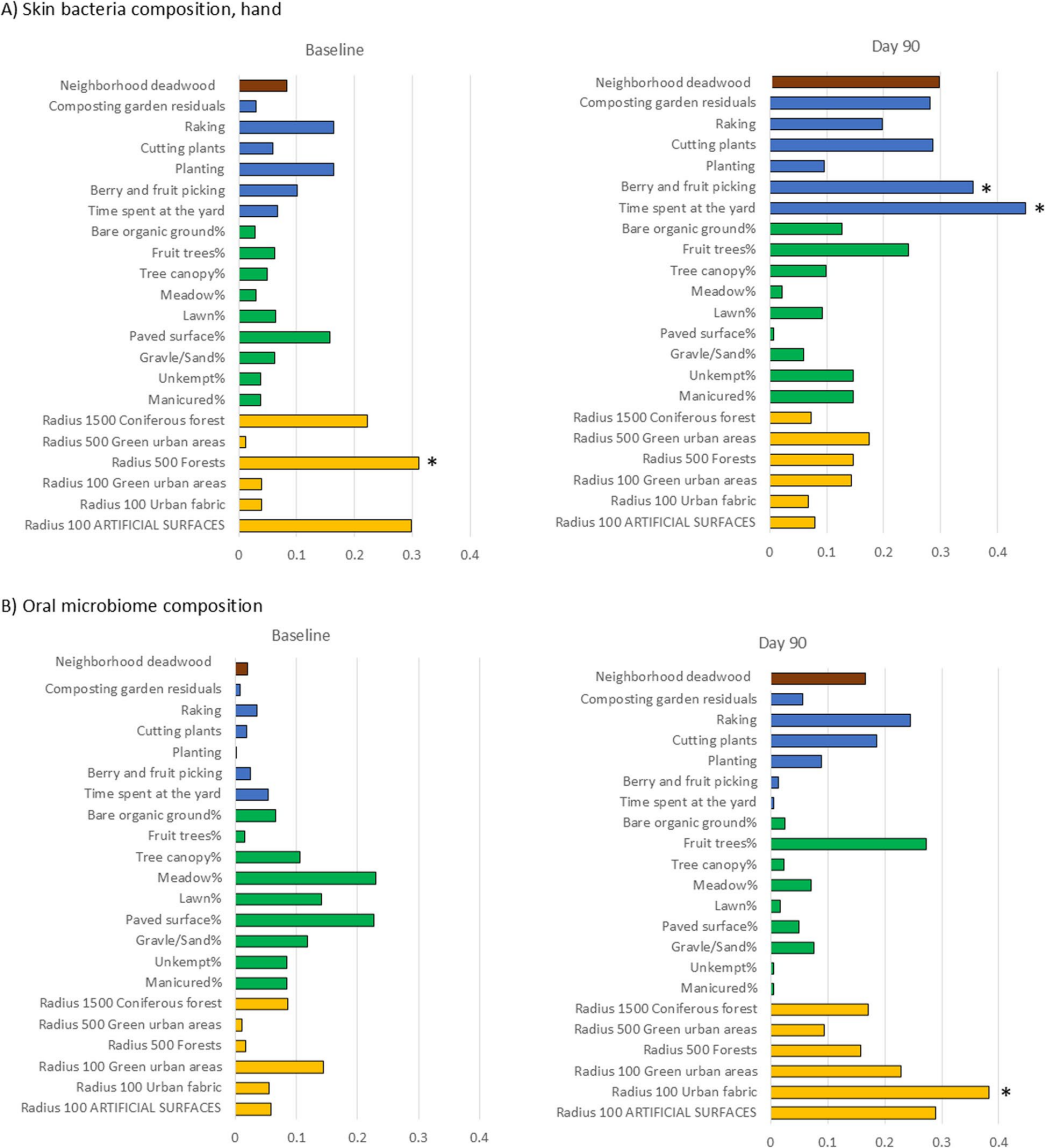
Significance and explained variance of 22 environmental variables modelled by EnvFit. Horizontal bars show the amount of variance (*R*^2^) explained by each environmental variable. **A** Skin bacteria compositions on the back of the hand using 16S rRNA sequencing data at ASV level and **B** oral microbiome compositions using metagenomic shotgun sequencing data at baseline and in the end of the study period (day 90). Environmental variables were applied separately to predefined variable groups and colored based on metadata group: brown = neighborhood deadwood (200 m radii around yards), blue = garden management practices, green = yard characteristics (percentage of yard area), yellow = land cover categories around home. *padj < 0.05. No association was observed between these 22 environmental variables and either forearm microbiota or oral functional gene pathways after adjusting for multiple testing (Figure S1)

At baseline, LMM models identified higher relative abundance of *Proteobacteria*, particularly gammaproteobacterial order Burkholderiales and family Neisseriaceae, on the forearm to be associated with higher polypore species richness (padj < 0.005, *R*^2^ = 0.78–0.82) and the amount of decaying deadwood in the neighborhood (padj < 0.04, *R*^2^ = 0.54–0.58; Table S3). However, these taxa were not detected by ANCOM-BC analysis. City was a significant confounding factor in LMM models because Helsinki had more deadwood and higher polypore species richness compared to Lahti (*p* and padj < 0.0001). At baseline, the presence of neighborhood deadwood explained 22% variation in forearm bacterial community composition, but the *p*-value was < 0.06 after multiple-testing correction (*p* = 0.024, padj = 0.059, *R*^2^ = 0.22; Table S4). Forest cover within a 500 m radius around participants’ homes explained 31% variation in skin bacterial community composition on the back of the hand at baseline (*p* = 0.007, padj = 0.04, *R*^2^ = 0.31; Fig. 1A and Fig. S1A). At the end of the study period, this association was not observed (*p* = 0.069). In contrast, urban fabric within a 100 m radius around yards explained the variation in oral microbial community composition at the end of the study period (*p* = 0.012, padj = 0.04, *R*^2^ = 0.38; Fig. 1B and Fig. S1D).

**Fig. 2:**
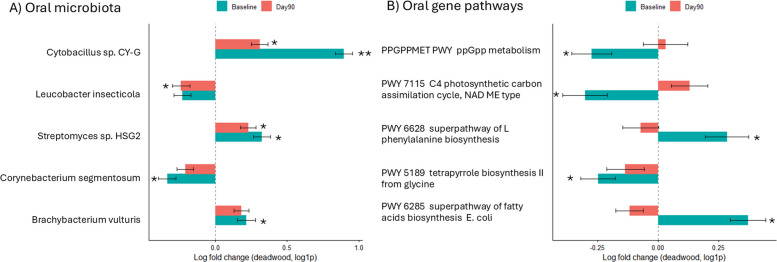
ANCOM-BC results for associations between neighborhood deadwood and the oral microbiome. **A **Taxonomic composition of the oral microbiota and **B** functional gene pathways. Effect sizes are shown as log fold changes for deadwood, with bars representing standard errors. Statistically significant associations after multiple-testing correction are indicated by *padj ≤ 0.05 and **padj ≤ 0.01

In the oral microbiome, ANCOM-BC identified several taxa associated with neighborhood deadwood at baseline and at the end of the study period, including three taxa with positive and two taxa with negative log fold changes when all study subjects were in the same model (*p* < 0.006, padj ≤ 0.5; Fig. 2A; Table S5A). When treatment groups were analyzed separately, these associations were not statistically significant after FDR correction, or they failed to pass the ANCOM-BC sample size screening criteria. In the analyses of oral functional gene pathways, the fatty acid biosynthesis superpathway (annotated to *Escherichia coli*) was positively associated with neighborhood deadwood at baseline (*p* = 0.004, padj = 0.05; Fig. 2B; Table S5B). This association was observed primarily among participants in the rewilding group and remained at the end of the study period within this group only (*p* ≤ 0.01, padj = 0.05). Although these associations were statistically significant after FDR correction, they did not meet the ANCOM-BC robustness criterion, indicating sensitivity to data perturbation. None of the observed associations between oral microbiomes and neighborhood deadwood were confounded by city (*p* > 0.05).

**Fig. 3:**
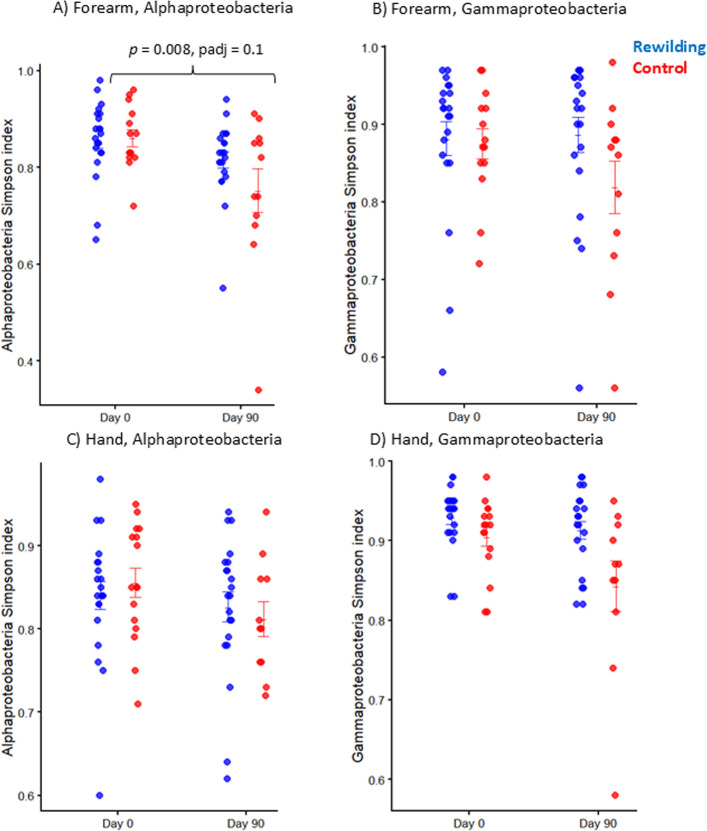
Skin Simpson diversity index of **A** Alphaproteobacteria and **B** Gammaproteobacteria on forearm, and **C** Alphaproteobacteria and **D** Gammaproteobacteriaon back of the hand between rewilding and control groups at baseline (Day 0) and in the end of the study period (Day 90). Error bars represent the mean ± standard error

### Yard management practices and yard characteristics explained variation in oral and skin microbial community composition

At the end of the study period, the frequency of berry and fruit picking (*p* = 0.01, padj = 0.04, *R*^2^ = 0.36) and time spent at the yard explained variation in skin bacterial community composition on the back of the hand (*p* = 0.004, padj = 0.02, *R*^2^ = 0.45, Fig. 1A; Fig. S1A). At baseline, no associations were observed between yard management practices and either skin microbial community composition (Fig. 1A) or the community composition of oral microbiomes (Fig. 1B). At the end of the study period, raking frequency showed a similar orientation as urban fabric in the ordination space, but unlike urban fabric, raking did not explain variation in the oral microbial community composition after adjustment for multiple testing (*p* = 0.05, padj = 0.28, *R*^2^ = 0.24; Fig. S1D).

Confounding factors including age, gender, BMI, pet ownership, and diet were not associated with oral microbiomes and skin bacterial profiles (Table S6A-D).

### Rewilding preserved skin bacterial diversity and richness to the end of the study period

Skin bacterial richness or diversity (Shannon and Simpson index) did not decrease in the rewilding group during the study period (Table S7A and B). In contrast, the alphaproteobacterial diversity showed a decreasing trend among the control group on the forearm, but this change did not remain statistically significant after adjustment for multiple testing (Simpson diversity index: *p* = 0.008, padj = 0.09, *R*^2^ = 0.19; LMM, Fig. 3; Table S7E). Skin alphaproteobacterial Simpson diversity index decreased from summer to the end of the study period also when both groups were analyzed together (Hand: *p* = 0.003, padj = 0.03, forearm: *p* = 0.004, padj = 0.05; LMM, Table S7C and F, respectively).

**Fig. 4:**
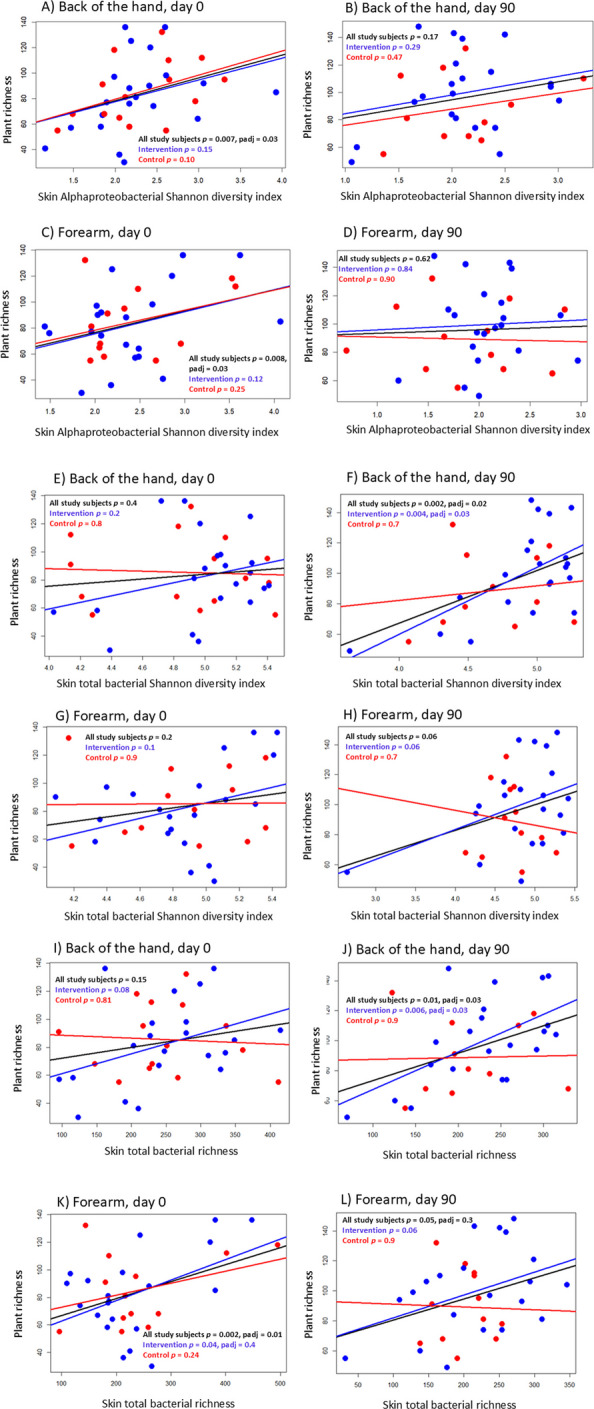
Correlation between yard plant richness and Alphaproteobacterial Shannon diversity index **A** on back of the hand at baseline and **B** fall, **C** on forearm at baseline and **D** fall, and between plant richness and skin total bacterial Shannon diversity index **E** on back of the hand at baseline and **F** fall, **G** on forearm at baseline and **H** fall, and between plant richness and skin total bacterial richness **I** on back of the hand at baseline and **J** fall, **K** on forearm at baseline and **L** fall

At the baseline, yard plant richness was directly associated with skin alphaproteobacterial diversity on the back of the hand (*p* = 0.007, padj = 0.03, *R*^2^ = 0.24; Table S8A; Fig. 4A) and on the forearm when all study subjects were included in the same LMM (*p* = 0.008, padj = 0.03, *R*^2^ = 0.23; Fig. 4B). At the end of the study period, plant richness was directly associated with total bacterial richness (*p* = 0.01, padj = 0.03, *R*^2^ = 0.18; Fig. 4J) and diversity on the back of the hand (*p* = 0.002, padj = 0.02, *R*^2^ = 0.24; Fig. 4F; Table S8A). When treatment groups were analyzed separately, the direct association between plant richness and skin total bacterial richness on the back of the hand (*p* = 0.006, padj = 0.03; Fig. 4J) and diversity (*p* = 0.004, padj = 0.03; Fig. 4F) were observed only within the rewilding group at the end of the study period (Table S8B). Treatments did not differ in yard plant richness at baseline or at the end of the study period (Table S1).

### Rewilding shifted commensal microbiota and oral functional gene pathways

**Fig. 5:**
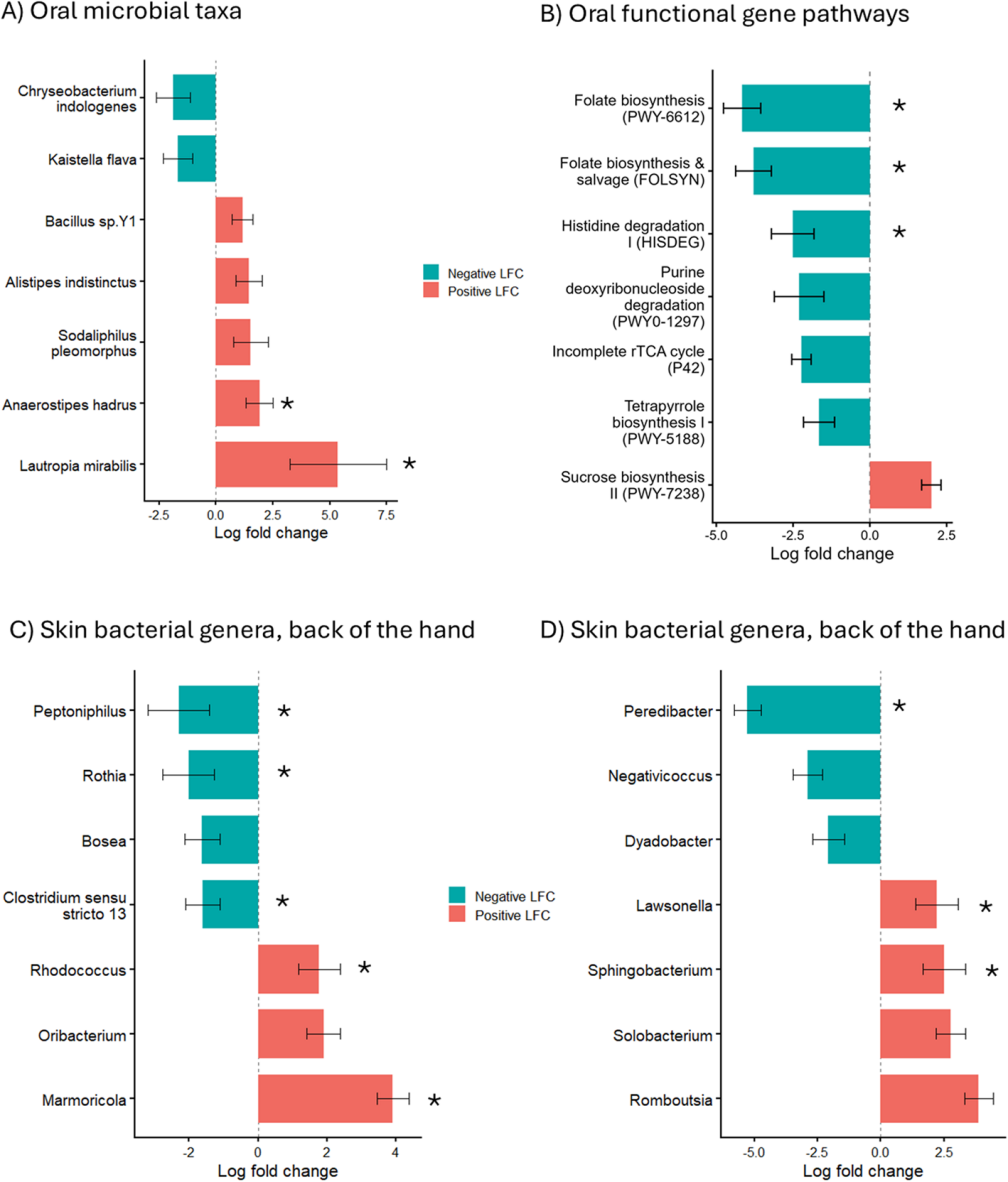
ANCOM-BC results for **A** taxonomic composition of the oral microbiota and **B** functional gene pathways, and **C** skin bacterial genera on the back of the hand and **D** forearm. Effect sizes are shown as log fold changes for treatment × time interaction, with bars representing standard errors. Statistically significant associations after multiple-testing correction are indicated by *padj ≤ 0.05

ANCOM-BC analysis revealed differential temporal responses of the oral microbiome to rewilding driven by a significant Treatment × Time interaction (padj ≤ 0.05; Fig. 5A and B; Table S9A and B). *Lautropia mirabilis* and *Anaerostipes hadrus* showed increased abundance in response to the rewilding (*p* = 0.01, padj = 0.05; Fig. 5A; Table S9A). In addition, superpathway of tetrahydrofolate biosynthesis (PWY 6612), superpathway of tetrahydrofolate biosynthesis and salvage (FOLSYN PWY) (*p* < 0.001, padj = 0.02), and L-histidine degradation I (HISDEG PWY) exhibited negative log fold changes associated with the Treatment × Time interaction (*p* = 0.002, padj = 0.05; Fig. 5B; Table S9B).

On the back of the hand, *Marmoricola* and *Rhodococcus* showed increased abundance, while *Peptoniphilus*, *Rothia*, and *Clostridium *sensu stricto showed decreased abundance in the rewilding group compared with the control group, as indicated by a significant Treatment × Time interaction (*p* = 0.01, padj ≤ 0.05; Fig. 5C; Table S9C). On the forearm, ANCOM-BC detected a positive log fold change for *Sphingobacterium* and *Lawsonella*, and negative for *Peredibacter* (*p* ≤ 0.01, padj < 0.05; Fig. 5D; Table S9D). However, these associations did not meet the ANCOM-BC robustness criterion, indicating sensitivity to data perturbation.

### IL-6 was associated negatively with richness of oral microbial species and functional gene pathways

At baseline, fruit trees (*p* and padj < 0.001, *R*^2^ = 0.82) and tree canopy percentage (*p* = 0.002, padj = 0.02, *R*^2^ = 0.32) were directly associated with salivary IL-6 when both treatments were analyzed in the same LMM model (Table S10C). At the end of the study period, higher total oral microbial, alpha- and gammaproteobacterial richness, and functional richness and diversity of gene pathways were associated with lower salivary IL-6 levels (Table 2; Fig. S2). Picking of berries and fruits was directly associated with salivary IL-10 (*p* = 0.002, padj = 0.02, *R*^2^ = 0.32) and IL-6 (*p* = 0.006, padj = 0.02, *R*^2^ = 0.52) in the rewilding group but not in the control group or at the baseline (*p* > 0.1; Table S10). When both treatments were in the same LMM model, potential confounding factors (age, gender, BMI, pet ownership, and diet) were not associated with immune markers (Table S6E and F).

**Table 2:** Linear mixed model (LMM) results at the end of the study period between salivary IL-6 and microbial richness and diversity indexes. LMM statistics are reported as *t* value, *p* value, permuted *p* value (5000 permutations), Benjamini-Hochberg adjusted *p* value and fixed R squared (*R*^2^) for the model

	***t* value**	***p* value**	**Permuted *p* value**	***p* adjust**	***R***^**2**^
Total richness	−3.076	**0.005**	**0.003**	**0.017**	0.273
Phyla Proteobacteria richness	−2.750	**0.010**	**0.004**	**0.017**	0.251
Class Gammaproteobacteria richness	−2.501	**0.019**	**0.007**	**0.017**	0.229
Class Alphaproteobacteria richness	−3.225	**0.003**	**0.003**	**0.017**	0.278
Functional richness	−2.910	**0.007**	**0.005**	**0.017**	0.255
Total Shannon	−2.223	**0.035**	**0.032**	0.054	0.158
Phyla Proteobacteria Shannon	−0.679	0.503	0.515	0.594	0.016
Class Gammaproteobacteria Shannon	−0.923	0.364	0.375	0.468	0.030
Class Alphaproteobacteria Shannon	−2.797	**0.009**	**0.009**	**0.020**	0.246
Functional Shannon	−2.894	**0.007**	**0.006**	**0.017**	0.245
Total Simpson	−0.993	0.330	0.318	0.433	0.036
Phyla Proteobacteria Simpson	0.147	0.884	0.891	0.891	0.001
Class Gammaproteobacteria Simpson	−0.559	0.581	0.612	0.656	0.011
Class Alphaproteobacteria Simpson	−1.232	0.228	0.147	0.220	0.057
Functional Simpson	−2.474	**0.020**	**0.013**	**0.025**	0.210

Note: Bold values indicate statistically significant differences (*p* < 0.05)

## Discussion

### Yard rewilding promotes commensal microbiomes and health-promoting ecosystem services

Our study is the first to show how rewilding of urban private yards with vegetation and decaying deadwood prevents the loss of microbial diversity on human skin and modifies oral microbiomes, functional gene pathways, and immune response. Our results also indicate that neighborhood deadwood and home yard plant richness are associated with previously human health-associated microbiomes [1, 15, 35].

Several factors underscore the significance of rewilding as a potential health-enhancing ecosystem service in our study. First, skin microbial diversity, quantified by species richness, Shannon and Simpson index, of skin proteobacterial classes remained stable among participants in the rewilding group only, even though gardening was practiced with similar frequency and time spent at the yards decreased in autumn in both groups. Since we specifically selected plant cultivars hosting rich bacterial community, these cultivars might have contributed to the observed difference. The same applies to gardening advice provided by our team; the rewilding group left plant litter to decompose naturally, which increases soil microbial diversity and resilience, and enhances nutrient cycle *on site* [36, 37]. The use of logs and chips, and biodiverse planting soil was designed to increase the overall and Proteobacterial exposure at the yards, and thus could contribute to the difference between the two study groups. Although environmental microbial diversity in the yards will be measured as part of broader, long-term research, the present manuscript does not evaluate the extent to which the rewilding intervention altered yard microbial communities. In earlier intervention trials [15, 38], skin Alpha- and Gammaproteobacteria have been associated with the higher frequency of T regulatory cells, which play a critical role in preventing autoimmunity [39]. Therefore, our findings are consistent with a potential role of biodiversity in supporting homeostasis and immunological self-tolerance, which need to be confirmed in longer-term studies.

Second, after sample size screening in ANCOM-BC2, we observed decreased skin relative abundance of *Peptoniphilus* that is commonly associated with diabetic skin and soft tissue infections [40], while the relative abundance of soil- and nature-associated bacterial genera increased on the skin of participants in the rewilding group. The latter genera, namely *Marmoricola*, *Rhodococcus*, and *Sphingobacterium*, are commonly detected in the rhizosphere and are known to promote organic matter turnover and nutrient cycling in natural environments [41–44]. Therefore, our findings suggest that home yard rewilding has increased participants’ direct contact with nature-based elements introduced into the yards, facilitating the transfer of environmental microbes onto the skin, which in turn may lead to a decrease in potential pathogenic bacteria on the skin.

Differences in the consequences of lifestyle also support the importance of rewilding. Only within the rewilding group, plant richness was associated with skin bacterial diversity at the end of the study period. Since we added several nature-based elements that plausibly enhanced the proteobacterial community on the skin, it is hard to distinguish the core reason for the differences in skin bacteria between rewilding and control groups. Nevertheless, our findings underline the importance of the quality of urban green space. Additional research is evidently needed to reveal the relative importance of each nature-based element. Today, the viable solution provided by the current study is to optimize them all.

### Changes in oral microbiomes and cytokines are in accordance with the biodiversity hypothesis

Our findings support the idea that environmental biodiversity exposure may influence commensal microbiomes and biological pathways implicated in host–microbe interactions. Specifically, several shifts observed in the oral microbiomes, functional gene pathways, and salivary cytokines are encouraging in the context of the biodiversity hypothesis.

The temporal effects in oral functional pathways associated with rewilding indicate a relative decrease over time in the rewilding group in tetrahydrofolate biosynthesis and salvage, and L-histidine degradation. The reduced abundance of tetrahydrofolate biosynthesis pathways may suggest decreased reliance on de novo microbial vitamin synthesis [45, 46], potentially arising from altered oral microbiota or changes in nutrient availability linked to increased environmental microbial exposure following home-yard rewilding. Since the degradation of L-histidine is interconnected with various metabolic pathways that are involved in the biosynthesis of essential amino acids [47], the observed shifts in L-histidine degradation pathway in oral microbiomes may have implications for cellular function, neurotransmission, immune regulation, and overall physiological balance and well-being. Because L-histidine degradation pathway I degrades L-histidine to L-glutamate, and because L-glutamate is associated with chronic inflammation [48, 49], the decrease in this pathway should contribute to reduction of proinflammatory signals. However, as sensitivity analyses indicated that the detected functional shifts may be sensitive to modeling assumptions and sample variability, studies with higher number of study participants and extended follow-up over multiple years are recommended. Such studies would determine for how long the functional shifts observed by us persist, and whether they have measurable consequences for immune-related health outcomes. Nevertheless, our study is the first to pave the way for microbially oriented large-scale rewilding studies in the context of the biodiversity hypothesis and planetary health.

A recent in vitro study suggested that L-histidine attenuates non-esterified fatty acid-induced inflammation by reducing Grb2-associated binder 2 expression, and eventually induces IL-6 expression [50]. In our study, the higher diversity of oral functional gene pathways was associated with lower IL-6 levels in saliva. Higher proinflammatory salivary cytokine levels have been associated with immune-mediated diseases, periodontal and tumor diseases [51, 52], stress [53, 54], and neuropsychiatric disorders [55]. Further support for the immunomodulatory role of rewilding comes from the observation that picking edible plants was associated with salivary cytokines in the rewilding group only. This association could relate to changes in plant-associated microbial exposure following rewilding.

We observed a differential longitudinal pattern in *Anaerostipes hardus*, with the rewilding group showing a greater increase over time relative to controls within the oral microbiota. *Anaerostipes hadrus* is a functionally important intestinal bacterium known for its role in butyrate production and associated probiotic effects [56]. While the functional role of *Anaerostipes hardus* in the oral cavity remains unclear, its established association with host health in the gut suggests that rewilding may promote the colonization or persistence of beneficial anaerobes beyond the intestinal ecosystem. The enrichment of *Lautrobia mirabilis* following rewilding further supports greater microbial stability and resilience of oral microbiota. *L. mirabilis* is a commensal member of the human oral microbiota and is frequently detected in healthy oral sites [57]. Several studies have reported a higher relative abundance of *Lautropia* species in periodontal health compared with disease, suggesting a potential association with eubiotic oral communities rather than dysbiosis [58–60]. Therefore, our finding could indicate that the rewilding promoted ecological conditions favoring commensal, health-associated taxa rather than organisms linked to inflammatory periodontal states. However, given the compositional nature of microbiome data and that this study did not assess health endpoints associated with these oral taxa, this interpretation should be made cautiously.

The current study, combined with previous human and animal research highlighting the benefits of soil microbiome exposure, such as improved gut homeostasis, enhanced immunity, prevention of allergies and autoimmune diseases, and reduced stress and anxiety [61, 62], suggests that microbiologically focused rewilding may influence biological and psychological processes relevant to health. In parallel with the observed shifts and differences between treatments, we did not observe enrichment of taxa associated with negative health impacts in the rewilding group. The extent and clinical significance of potential health benefits remain to be determined in future studies.

### Neighborhood deadwood as an indicator of microbially rich urban environments linked to commensal microbiomes

The hypothesis that decaying wood is an important factor modulating commensal microbiota of urbanities is supported by our study. First, proteobacterial taxa on the skin were directly associated with the decaying deadwood in the neighborhood. Since decaying deadwood is used as a biodiversity indicator and exists in boreal nature together with other decomposing plant matter and a vivid moss layer [25, 26], the finding highlights the importance of plant litter and biodiversity in general. Interestingly, the same bacterial taxa that we found abundantly on skin, i.e., Alpha- and Gammaproteobacteria and *Burkholderiales*, are rich in decaying wood [63–68]. However, the findings describe associations at higher taxonomic levels, and without species-level resolution, no conclusions regarding health implications of individual species can be drawn.

Interestingly, several oral microbial taxa were consistently associated with the amount of neighborhood deadwood both at baseline and at the end of the study period. This suggests a stable relationship between local environmental characteristics and the oral microbiota. Importantly, deadwood itself is unlikely to be the direct exposure source. Rather, it functions as an indicator of urban environments where natural ecological processes are allowed to occur, such as unmanaged or semi-natural green space fragments. These environments typically support diverse plant and fungal communities, including dwarf shrubs, berry-producing plants, and fungi, which are characteristic of habitats where deadwood accumulation is possible.

A plausible mechanism underlying this association is behavioral exposure during nature use. Urban residents that visit nature in Finland tend to be in contact with microbiota associated with decaying organic matter via their hands when touching soil and plants. Specifically, foraging-related behaviors, such as collecting, tasting, or consuming natural plants while in nature, may facilitate microbial transfer from the environment to the oral cavity. Other exposure pathways could be airborne microbial transfer [69]. Birch leaves, wood sorrel (*Oxalis acetosella*) leaflets, rock fern roots, and bilberry flowers are examples of commonly encountered edible wild plant parts that grow in places with a high amount of decaying deadwood. Therefore, they could act as vectors for environmental microbes. Because these edible wild plants are directly ingested or handled immediately prior to ingestion, such exposures would be expected to be reflected in skin and oral microbial profiles. The taxa associated with neighborhood deadwood, including *Cytobacillus* sp. CY-G and *Streptomyces* sp. HSG2, are consistent with this interpretation. Both genera are commonly associated with soil and decaying organic matter and are well adapted to environments characterized by plant litter decomposition and microbial competition [70–72].

In contrast to the relatively stable associations observed between neighborhood deadwood and specific oral microbial species, associations with predicted oral functional gene pathways appeared less consistent over time. At baseline, the fatty acid biosynthesis superpathway annotated to *Escherichia coli* showed a positive association with neighborhood deadwood. However, this relationship was evident primarily among participants in the rewilding group and persisted at the end of the study period only within this group. This suggests that functional responses of the oral microbiome may be more sensitive to behavioral, such as diet, or environmental modification than taxonomic composition alone. The fatty acid biosynthesis superpathway represents a core metabolic function involved in membrane synthesis and cellular growth and is broadly conserved across bacteria [73]. In the oral cavity, variation in the relative abundance of this pathway may reflect shifts in microbial growth dynamics or transient enrichment of environmentally derived bacteria rather than stable functional reprogramming of the resident oral microbiota [74]. The group-specific persistence of this association in the rewilding group is consistent with the interpretation that rewilding or sustained nature exposure may be required for functional signals to remain detectable over time.

### Contrasting garden management types shape skin microbial composition

Interestingly, coverage of unkept garden area showed contrasting alignments with skin bacterial community along principal coordinates axes than a high coverage of manicured garden area and lawn (Fig. S1B). This indicates that the management of garden explains the variation in skin bacterial community compositions. In several earlier studies [2, 28], buildings and paved surfaces were associated with an increased risk of immune-mediated diseases. The current study thus provides an explanation for how paving and removal of plant litter may be connected to human microbiomes associated with inflammatory signals. Unfortunately, the low concentration of microbial DNA in skin swabs did not allow metagenomic sequencing.

Even though previous studies pointed out the importance of vegetation for health-associated microbiota transferred indoors [75–77], our study is the first to separate the roles of plant richness versus neighborhood deadwood abundance. Deadwood and plant residues seem to play a role in the health-associated microbiomes comparable to that of living vegetation. Until now, vegetation indices were a major if not the sole indicator of biodiversity in studies that tested how biodiversity is related to immune-mediated diseases [22]. Our study opens a new avenue for microbiome research; studies focusing on health-associated ecosystem services should investigate neighborhood deadwood and plant residues in parallel with live vegetation.

The lack of associations between microbial compositions and age, gender, BMI, or diet in this study differs from findings reported in previous studies [8]. In our study, participants had relatively similar dietary patterns and BMI. In addition, the even age distribution of participants in both study groups may have masked potential age-related differences. This plausibly reduced variability and limited their detectable effects, while at the same time minimized the role of confounding factors and thus allowed a more focused assessment of how yard rewilding and environmental characteristics relate to the commensal microbiota. In this context, the study design was well suited to examine environmental variables despite the relatively small sample size. In contrast, environmental exposures such as berry-picking activity and tree canopy cover varied more substantially between participants and may therefore have been detectable in the analysis.

The current study pioneers the rewilding of private yards and has therefore its limitations. First, the sample size (21 participants in the rewilding group vs. 15 controls) may have limited the ability to detect smaller effects in secondary and exploratory analyses, as the study was powered primarily for the predefined primary outcome. Effect size estimates used for sample size calculations were derived from the most comparable prior biodiversity intervention studies, as no previous studies exist with identical or even similar rewilding design. Second, the study was a non-blinded, non-randomized, non-crossover intervention, which may increase susceptibility to residual confounding and limits causal inference. Third, although all study participants had gardening backgrounds, self-selection bias cannot completely be excluded, which may limit the generalizability of the findings to the broader urban population. If we had the option to do the trial among average urbanites, the changes observed could be even stronger.

Fourth, the control condition included commonly used garden management products, which may have minor effects on environmental microbial communities and therefore cannot be considered a completely inert control. This design choice was necessary to reflect realistic gardening practices and to ensure participant engagement but should be considered when interpreting contrasts between groups. Fifth, because microbiome sequencing data are compositional [78], analyses based on relative abundances describe proportional shifts in community composition rather than absolute changes in microbial load, and results should be interpreted accordingly. Finally, plant abundance was estimated using a standardized categorical scoring system rather than biomass or surface area, which may not fully capture differences in plant size or potential microbial exposure. Taken together, these findings should be viewed as hypothesis-generating and provide a foundation for larger future studies that can more precisely quantify rewilding effects.

## Conclusions

This study showed that lifestyle changes resulting from urban rewilding induce shifts in the commensal microbiomes and biological pathways, including changes in immunomodulatory oral functional gene pathways and cytokine levels. Decaying deadwood in the surrounding neighborhood as well as home yard plant richness may both induce shifts in commensal microbiota. Hence, our findings support the notion that urban green spaces permitting natural decay processes may shape human commensal microbiomes. Because neighborhood deadwood served primarily as an indicator for biodiversity, its association with commensal microbial composition underscores the relevance of microbially rich urban nature for environmental–human microbial exchange. The importance of a biologically diverse living environment in immunomodulation underscores the need to allow natural decaying processes to occur and to apply a microbially focused rewilding approach in urban green spaces. Future studies should use manually inventoried and satellite-data-based deadwood/plant litter indices in parallel with vegetation indices when estimating the associations between biodiversity and immune-mediated diseases. Our findings provide an incentive for future urban rewilding approaches with microbially enriched organic soil, plants, and decaying deadwood to support human microbiomes and health.

## Materials and Methods

### Study group

Thirty-six volunteers aged between 33 and 70 participated in the study (Table 1). The participants were recruited to two groups. The intervention group, called the rewilding group, consisted of volunteers who gave permission to diversify yard herbaceous and woody species community and were interested in following the yard management instructions described below in the “Experimental design” section. The control group continued gardening as they had done before. Study subjects were recruited from April 2022 to June 2022 in three metropolitan regions in Finland; the Helsinki metropolitan region (660,000 inhabitants) lies on the south coast of Finland, Lahti (120,000 inhabitants) 100 km north, and Turku (480,000 inhabitants) 150 km west of Helsinki. All these urban housing areas face challenges in preserving green space amid the built environment. Participants were recruited by sending an invitation letter to households in urban detached-house areas in the Helsinki, Lahti, and Turku regions. 

The medical exclusion criteria included immune deficiencies, a disease affecting immune response, cancer diagnosis within the last year or ongoing cancer treatment. Other exclusion criteria included incompetency and living outside city area, defined as locations with less than 90% coverage of the CORINE land-use category Artificial surfaces: Urban fabric. Eligible participants were required to live in either a detached house or a row house. All participants provided a written, informed consent in accordance with the Declaration of Helsinki.

The participants filled out questionnaires at baseline in July before rewilding and three months later in early November 2022. Questionnaires collected detailed background information regarding diet, nature visits, medication use, yard management practices, and activities at the yard (Supplementary methods).

### Outcome measures and sample size

The primary outcome measure for sample size estimation was the difference in skin gammaproteobacterial richness between the rewilding and control groups. Secondary outcomes were as follows: (1) between-group differences in richness, diversity and relative abundance of skin and oral microbiota; (2) between-group differences in oral microbiome functional gene pathways; and (3) between-group differences in salivary cytokine levels (IL-6, IL-10). Exploratory analyses assessed associations between environmental factors, yard management practices, activities at the yard, salivary cytokine levels, and microbial measurements. Sample size calculations for the number of study participants were based on previously reported effect sizes for biodiversity-related differences in skin Gammaproteobacterial diversity and associated immune markers [2, 14, 15, 38]. Previous biodiversity intervention studies have demonstrated that increases in skin Gammaproteobacterial diversity are associated with changes in immune markers, supporting its use as an indicator of biodiversity-related immune modulation [15, 38]. Assuming three city-level clusters, a coefficient of variation of 0.2, a significance level of 0.05, and 80% power, the required sample size was estimated as 15 participants per treatment group (see Supplementary Methods for details).

## Experimental design

### Rewilding group

An urban planner–architect Laura Uimonen within the research team designed the yards of the rewilding group using a predefined set of nature-based elements. We applied the nature-based elements to the rewilding sites according to yard-specific plans in July 2022. Nature-based elements included berry bushes, fruit trees, perennial yard plants, annual herbs, meadow transplants (1 m^3^), cultivation boxes (*n* = 3), organic mulch materials, decaying deadwood, leaf compost, and organic soil with high microbial diversity. We selected berry bushes, fruit trees, and yard plants based on previously reported high microbial diversity and richness in leaf buds [79] (Table S11).

Yards in the study varied substantially in size, existing vegetation, and garden age, ranging from plant-diverse cottage gardens with mature fruit trees to newly built detached houses with small lawns. Consequently, all participants did not receive identical plant species or planting densities. Rewilding aimed to enhance plant species diversity. The amount and selection of added plant species for each yard were based on a pre-rewilding inventory, and only plant species absent prior to the rewilding were introduced, while considering site-specific growing conditions. The number of added perennial plant species per rewilding yard varied from 6 to 38. We implemented nature-based elements with minimal disturbance to existing trees, shrubs, and perennials. We primarily installed these elements on lawns or bare soil and used them to densify existing flowerbeds. We positioned plants, cultivation boxes, and decaying deadwood near walkways, doors, and terraces.

We introduced meadow flowers to each rewilding yard using a 1 m^2^ transferable meadow plot (Grown at Terolan Taimitarha garden center, Tuulos, Finland), which included yarrow (*Achillea millefolium)*, maiden pink (*Dianthus deltoides*), wild strawberry (*Fragaria vesca*), lady’s bedstraw (*Galium verum*), oregano (*Origanum vulgaris*) and wild thyme (*Thymus serpyllum).* We selected the planting soil based on previous studies comparing commercially available soils and selected the ones that included high microbial richness (observed species richness over 4000) [12, 80, 81]. The soil consisted of composted materials (wood fiber, chicken manure, bark grit, *Sphagnum* moss, biochar, light growing peat) and was manufactured by Biolan Oy (Eura, Finland; trade names Musta Multa, Puutarhan Sammalmulta, Turpeeton Puutarhamulta).

Study subjects in the rewilding group received three cultivation boxes per yard. Cultivation boxes were built from two stacked large pallet collars (120 × 80 cm), giving a volume of 0.38 m^3^. One cultivation box was planted with herbs, including lovage (*Levisticum officinale*), chives (*Allium schoenoprasum*), thyme (*Thymus vulgare*), chervil (*Anthriscus cerefolium*), peppermint (*Mentha piperita*), and parsley (*Petroselinum crispum*). The second cultivation box per yard was left empty to allow participants to grow annual edible plants of their own choosing. The third cultivation box contained decaying deadwood in the form of three 1-m silver birch (*Betula pendula*) (Fig. S3). KÄÄPÄ Biotech (Lohja, Finland) had inoculated the logs with lion’s mane (*Hericium erinaceus*) and shiitake (*Lentinula edodes*) fungi two years prior to the rewilding. Decaying deadwood was also transferred to each rewilding yard in the form of wood chips composed of birch, aspen, and oak. A total of 1 m^3^ of these wood chips, provided by KÄÄPÄ Biotech, was used in framing planting areas and walking paths, and as mulch in plantings and in play areas.

The rewilding group received leaf compost and we gave written instructions to the rewilding group on yard management practices and how to recycle organic matter and nutrients on site. These instructions included reducing raking, chopping autumn leaves onto lawns, and composting plant material. Additional guidance was provided on the management of meadow plants, perennials, bushes, and trees, including instructions on protecting trees and bushes from rodents and winter frost, as well as on converting lawn areas into meadow vegetation.

To improve the completeness of reporting, and ultimately the replicability of intervention, we used the Template for Intervention Description and Replication (TIDieR) checklist and guide that is an extension of the CONSORT 2010 statement [82].

Intervention lasted 90 days from July 2022 to November 2022.

### Control group

The control group’s yards were not modified. The control group received inorganic fertilizers, moss remover, and ant control insecticide (Myrr®; active ingredient imidacloprid 0.03% w/w).

### Skin swab collection and microbial analyses

Participants collected skin swabs at baseline in July 2022 before rewilding and three months later in early November 2022 following written and illustrated instructions provided by the research team. At each sampling time point, participants used two separate sterile cotton-wool swabs (deltalab), wetted in 0.1% Tween® 20 in 0.15 M NaCl, to sample two sites on the dominant hand: (1) back of the hand (4 × 4 cm area), (2) forearm (5 × 5 cm area). Each site was swabbed for 10 s using its own swab. After collection, participants stored the swabs in their home freezers at −20 °C. Within 1 week, the research team transferred the swabs to −80 °C for long-term storage. Two skin sites were sampled to distinguish microbial changes at a site with direct and frequent contact with soil and vegetation (the back of the hand) from a site with more limited direct soil contact (the forearm). This design allows assessment of whether rewilding-related environmental exposure results in localized microbial changes restricted to direct contact surfaces or whether changes are also detectable at less directly exposed skin sites. Previous studies have demonstrated pronounced microbial shifts on hand surfaces following soil contact [12, 15, 38]; inclusion of the forearm enabled evaluation of the spatial extent of these effects.

Skin microbiota was characterized using 16S rRNA gene amplicon sequencing. This approach was selected because skin swab samples typically yield low amounts of microbial DNA, which can limit the reliability of shotgun metagenomic sequencing [83]. DNA was extracted with DNeasy® PowerSoil® Pro Kit (Qiagen, Hilden, Germany) according to the manufacturer’s standard protocol. PCR was performed for the V4 region within the 16S rRNA gene (three technical replicates from each sample) using 505 F and 806R primers [84]. Negative controls for DNA extraction, PCR, and sequencing (sterile water) and the ZymoBIOMICS™ Microbial Community Standard (Zymo Research Corp., Irvine, CA, USA) were included in DNA extraction and PCR to ensure the quality of the analysis. Paired-end sequencing of the amplicons (2 × 300 bp) was performed on an Illumina Miseq instrument using a v3 reagent kit.

Sequencing yielded a total of 2,261,702 reads, with a mean of 16,155 ± 6985 (standard deviation) reads per sample**.** Sequencing depth was comparable between treatment groups: rewilding group 15,514 ± 6799 and control group 17,729 ± 6813. No samples were discarded, as all samples met the minimum sequencing depth and quality thresholds required for analysis. Sequencing depth was sufficient, with a mean Good’s coverage of 0.99 ± 0.001 across samples.

Raw paired-end sequence files were processed into amplicon sequence variants (ASVs) using DADA2 (v1.26) [85] with the non-redundant Silva database version 138 [86]. ASVs detected in negative controls were evaluated during quality control and excluded from downstream analyses, while the mock community was used for quality assurance. To adjust for the different sequence read depths, the data were transformed to proportions by dividing the reads for each amplicon sequence variant (ASV) in a sample by the total number of reads in that sample [87].

### Saliva sample collection, metagenome shotgun sequencing and cytokine measurements

Saliva samples for microbial analyses were collected twice, at baseline before the rewilding and again 90 days later. At each sampling time point, participants collected saliva immediately after waking up sequentially holding three sterile cotton swabs in the mouth for 40 s each during the same sampling session. Thereafter, saliva samples for cytokine analysis were collected into empty collection tubes with no additives by passive drooling before eating or brushing teeth. The samples were subsequently frozen at −20 °C and within a week transferred to −80 °C. 

Salivary microbiota was analyzed using shotgun metagenomic sequencing because shotgun sequencing enables comprehensive profiling of microbial communities beyond bacteria, including fungi and viruses, as well as functional potential [83]. Whole metagenome shotgun sequencing was performed at FIMM – Institute for Molecular Medicine Finland as part of the Helsinki Institute of Life Science HiLIFE at the University of Helsinki. Libraries generated from total genomic DNA extracted from saliva were sequenced on the Novaseq S1/300c (Illumina) platform using the 2 × 150 bp paired-end read protocol. Negative controls (sterile water) and a mock community standard were sequenced alongside biological samples and used for quality control.

The quality of the sequencing reads was assessed with FastQC (v0.11.9) and low-quality, ambiguous sequences, adapters and contaminants detected in negative controls were removed using Cutadapt (v4.6). Human-derived sequences were removed during the downstream bioinformatic analysis. After quality control, sequencing yielded a total of 5,410,946 reads, with a mean of 85,888 ± 43,669 (standard deviation) reads per sample**.** Sequencing depth was comparable between treatment groups: rewilding group 84,331 ± 48,276 and control group 86,724 ± 41,596. Sequencing depth was sufficient, with a mean Good’s coverage of 0.99 ± 0.003 across samples. All samples met the minimum sequencing depth and quality thresholds required for analysis.

Reads were assembled into contigs prior to taxonomic assignment using MEGAHIT (v1.2.9). Taxonomic profiling of the final read set was performed using Kraken2 (v2.12) [88], while functional profiling was carried out using HUMAnN3 (v3.7) [89]. Whole-genome shotgun data were adjusted for differential sampling effort using centered log-ratio (CLR) transformation [78].

For cytokine measurements, samples were thawed and IL-6 and IL-10 concentrations were determined with a custom U-plex kit and MESO Quickplex SQ 120 by Meso Scale Discovery (Rockville, MD, US), according to the manufacturer’s instructions. Samples were used undiluted.

### Land cover category classification

We analyzed the land cover categories surrounding the dwellings of the participants with the preclassified Coordination of Information on the Environment (CORINE) Land Cover 20-m raster [90]. The spatial analyses were conducted using a custom Python program developed by the author (D.C.). The resulting datasets were subsequently visualised and validated in QGIS. Land cover data were used to characterize participants’ living environments and to apply exclusion criteria, with urban residency defined as locations having ≥ 90% coverage of the CORINE land-use category *Artificial surfaces*. We performed the land cover analysis using the data from the year 2018. The calculations used in this study included five buffer zones, 100, 500, 1000, 1500, and 2500-m radii around dwelling coordinates. Multiple buffer zones were used to capture environmental characteristics at spatial scales relevant to different types of daily exposure, ranging from immediate surroundings of the dwelling to the broader residential environment.

### Vegetation, deadwood, and polypore fungi inventories

We did a vegetation inventory in each private yard of the study subjects in June, before the rewilding intervention. Vegetation surveys were conducted once at baseline to characterize the stable environmental context of each area, with additional information recorded on plant species introduced as part of the rewilding intervention. Plant richness was calculated as species richness**,** defined as the total number of distinct plant species recorded within each survey area. The abundance of each plant species was estimated at scale:

1. Very scarce: fewer than 5 individual plants
2. Couple of individuals: more than 5 individual plants but no established populations
3. Few established populations: fewer than 3 distinct plant populations
4. Many established populations: more than 3 distinct plant populations
5. Very abundant: Dominates a specific growing area (e.g., lawn, meadow, or planting bed)
6. Dominant: Dominates across the whole study area (i.e., the whole yard)

We recorded background information that included yard area size, age (defined as the time since establishment of the house and associated yard), yard state (classified as natural, semiwild, manicured, or unkept based on management intensity), and estimated cover of mosses on the soil surface. In addition, land cover composition was recorded by visually estimating the percentage cover of predefined land cover categories within each yard, including gravel/sand, paved surfaces (tiles, concrete, asphalt), lawn, dry meadow, meadow, tree canopy cover, ditch, flowers, or other perennials, ornamental shrubs, berry bushes, rock, rock yard, fruit trees, shoreline or wetland, forest stand, idle land, bare organic ground.

To provide indicators of habitat naturalness in the landscape surrounding the private yards, we inventoried neighborhood deadwood and fruiting bodies of wood-inhabiting polypore fungi [29]. These inventories were conducted in the fall, when fruiting bodies of polypore species are readily detectable. Neighborhood deadwood and fruiting bodies of wood-inhabiting polypore fungi [27] inventories were done within 200 m radii around the yards, excluding the private yard area. A 200 m radius was selected to capture neighborhood-scale deadwood availability and fungal habitat that are relevant to participants’ broader environmental exposure, while minimizing the influence of very localized or transient features. These inventories represent environmental conditions and do not include the deadwood added as part of the rewilding intervention. The survey was restricted to polypore species with perennial or persistent annual fruiting bodies. Because neighborhood deadwood availability and the fruiting bodies of these species are structurally stable over the time scale of the study, inventories were conducted only once per area. Neighborhood deadwood inventories encompassed all green areas and trees within public areas. We inventoried all dead trees with ≥ 15 cm diameter at breast height (1.3 m) and other pieces of coarse woody debris with ≥ 15 cm basal diameter and ≥ 1.3 m length. For each dead tree and piece of deadwood, we recorded tree species and decay class [91] and occurrences of polypore fruiting bodies. In addition, polypore fruiting bodies were recorded from living trees with internal decay or attached dead branches. Decaying deadwood in our study represents decay class ≥ 2 in Renvall (1995) classification: Wood fairly hard and knife penetrates ca. 1–2 cm into the wood [91].

### Statistical analyses

We did the statistical tests with R v4.3.1 [92] and with *vegan* (v2.6–4) [93], *lme4* (v1.1–35) [94], *phyloseq* (v1.48.0) [95], and *ANCOMBC* (v2.6.0) [96] packages. Shannon and Simpson diversity indices were calculated using the *diversity* function and species richness with the *specnumber* function in the *vegan* package.

Differential abundance was assessed using ANCOM-BC2 [96], which has been shown to yield results that are more consistent across studies and align well with consensus findings across multiple differential abundance methods [97]. Taxa passing sample-size screening (passed_ss = TRUE) with FDR-adjusted q-values ≤ 0.05 were considered statistically significant. Robustness to data perturbation was evaluated using the ANCOM-BC robustness criterion (diff_robust). Microbial features (skin genera, oral species, and functional gene pathways) were used as a response variable. Explanatory variables included neighborhood deadwood abundance (log-transformed), timepoint, treatment, and city (included as an adjustment covariate), with all lower-order and three-way interaction terms between deadwood abundance, timepoint, and treatment. To account for repeated measurements within participants, a subject-specific random intercept was included in the model. Microbial features present in at least 10% of samples and total count ≤ 50 were retained for analysis.

We used Linear mixed-effects models (LMMs; *lmer* function in the lme4 package) to analyze temporal changes in microbial diversity and salivary cytokine levels. We used observed species richness, Shannon and Simpson diversity indexes, or cytokine level as response variables. Treatment and timepoint were included as explanatory variables, with timepoint representing repeated measurements within individuals. We used individual participants nested within city as random effects. This modelling framework allowed assessment of rewilding-related changes while accounting for baseline differences among participants.

We analyzed associations between microbial variables and living environment characteristics using LMMs with microbial measurements (relative abundance, richness, and diversity indices) as response variables, land cover categories, deadwood quantity, or polypore species richness as explanatory variables, and city included as a random effect. We report baseline associations to characterize pre-rewilding environmental conditions, whereas temporal analyses evaluate changes over time.

We determined the changes and difference between treatments in questionnaire data using generalized linear models (GLM) with binomial distributions [function *glmer* in *lme4* package]. We used questionnaire responses as response variables and treatment and timepoint as explanatory variables. Associations between yard characteristics and salivary cytokine levels were examined using GLMs with quasi-Poisson error distributions, with cytokine levels as response variables and yard characteristics as explanatory variables.

We analyzed skin and oral microbial compositions (response variable) with Permutational Multivariate Analysis of Variance (PERMANOVA, function *adonis2* in *vegan* package) with Bray–Curtis dissimilarities [98]. Treatment and timepoint were used as explanatory variables. PERMANOVA analyses were conducted separately for skin (ASV level) and saliva (species level) microbial communities using Bray–Curtis dissimilarities. Differences between treatments were assessed separately at baseline and at follow-up**,** and temporal changes in microbial community composition were evaluated within each treatment group by comparing baseline and follow-up samples. We used principal coordinates analysis (PCoA) based on the Bray–Curtis dissimilarities (*cmdscale* function) for visualization.

To evaluate how environmental variables (explanatory variables) explain variation in skin and oral microbial community composition (response variable), we performed principal coordinates analysis (PCoA) with Bray–Curtis dissimilarities and used the *envfit* function in the *vegan* package to assess the strength and orientation of environmental factors in multivariate ordination space.

We verify the P-value approximations with permutation tests (*lmperm* in *permuco* package) (5,000 permutations) [99]. We considered the statistical tests significant when permuted (p_perm) or Benjamini–Hochberg adjusted *p*-value (padj) was < 0.05 level. 

## Supplementary Information

Supplementary Material 1.

## Data Availability

Skin bacterial sequence data were accessioned into the Sequence Read Archive (BioProject ID: PRJNA1102262). The plant, deadwood, polypore inventory data and oral microbial taxa and functional gene pathways data are available in the Fairdata IDA (ida.fairdata.fi) continuous service [(10.23729/fd-e2737a9f-b50e-32b8-a903-937c8241c730)] and shotgun sequences [(**https:/doi.org/10.23729/fd-461a2589-7d56-31c0-ae58-774b72982263**)]. R scripts implementing ANCOM-BC and linear mixed-effects models are available in a public GitHub repository: [(https://github.com/MarjaIR/Yard_rewilding_microbiome_analysis)]. All other data needed to support the conclusions of this manuscript are included in the main text and supplementary appendix. The sensitive data that support the findings of this study are available from the Natural Resources Institute Finland, but restrictions defined in the General Data Protection Regulation (EU 2016/679) and Finnish Data Protection Act 1050/2018 apply to the availability of these data, and so are not publicly available. Data are available upon reasonable request and with permission from the ethical committee of the local hospital district (Helsinki and Uusimaa Hospital District, Finland). As shotgun sequences are controlled access, researchers must request access to the sensitive data. Requests for information addressed to the Natural Resources Institute Finland (Luke) in accordance with the Publicity Act (www.finlex.fi) (621/1999) should be sent separately to the registry office (email: kirjaamo@luke.fi).
