# Supplementary material for: Neighborhood deadwood and yard rewilding modulate commensal microbiomes and inflammatory signals among urbanites
Source: Microbiome. 2026 May 15;14:181. doi: 10.1186/s40168-026-02413-w (PMC13374267; doi:10.1186/s40168-026-02413-w)
Supplement: Supplementary file 2 — Supplementary Material 1. [file 40168_2026_2413_MOESM1_ESM.docx]

Supplementary materials

Supplement to: Marja I. Roslund, Laura Uimonen, Laura Kummola, Damiano Cerrone, Ann Ojala, Anna Luukkonen, Ella Holopainen, Aku Korhonen, Reijo Penttilä, Mika Saarenpää, Martti Venäläinen, Hanna Haveri, Juho Rajaniemi, Olli H. Laitinen, Aki Sinkkonen, and the BIWE research group. **Neighborhood deadwood and yard rewilding modulate commensal microbiomes and inflammatory signals among urbanites**

**Supplementary materials**

[Supplementary methods 3](#_Toc220921199)

[1. Questionnaire data 3](#_Toc220921200)

[2. Sample size calculation for study participants 3](#_Toc220921201)

[Supplementary results 4](#_Toc220921202)

[Table S1. T test results for yard characteristic between control and rewilding groups. 4](#_Toc220921203)

[Table S2. Generalized linear mixed model (GLMM) results for yard activities and garden management practices, animal contacts, and nature visits A) between treatments on day 90 (fall), B) within rewilding and C) control groups between baseline (summer) and day 90 (fall). 5](#_Toc220921204)

[Table S3. Linear mixed model (LMM) results for correlation between skin microbiota on the forearm and polypore species richness and decayed deadwood at A) baseline (summer) and B) day 90 (fall). 7](#_Toc220921205)

[Table S4. Correlation between beta diversity of skin bacteria on A) back of the hand and B) forearm, and decaying deadwood amount (N) and polypores at baseline and day 90. Beta diversity is reported for total bacterial community level (ASV). 8](#_Toc220921206)

[Table S5. Age, gender, body mass index (BMI), pet ownership, and diet association with A) salivary microbial species, and B) functional gene pathways, and C) skin bacteria on the back of the hand, and D) forearm, and cytokines E) IL-10 and F) IL-6.). 9](#_Toc220921207)

[Table S6. Linear mixed model (LMM) results between baseline and fall in skin bacterial richness and diversity on the back of the hand within A) rewilding and B) control treatment, and with C) all study subjects in the model, and on the forearm within D) rewilding and E) control treatment, and with F) all study subjects in the model. 11](#_Toc220921208)

[Table S7. Linear mixed model (LMM) results for correlation of yard plant richness with skin bacterial richness and diversity indices on the back of the hand. Analyses were done with A) all study subjects, and B) rewilding group study subjects, and C) control study subjects at baseline and 90-days post-intervention. LMM statistics are reported as t value, p value, Benjamini-Hochberg adjusted p value and R squared (R^2^). 13](#_Toc220921209)

[Table S8. Linear mixed model (LMM) results for Mycobacteriaceae, Leuconostoc and L-histidine degradation I pathway in saliva A) within rewilding and B) control treatments between baseline (day 0) and day 90, and C) change difference between treatments. 14](#_Toc220921210)

[Table S9. Correlation between salivary cytokines (IL-6 and IL-10) and yard characteristics, and berry picking within A) rewilding and B) control treatment, and with C) all study subjects in the GLM model. 15](#_Toc220921211)

[Table S10. Garden plants were chosen based on their bud microbial richness and Shannon-Wiener and Simpson diversity indexes. 16](#_Toc220921212)

[Figure S1. Principal coordinated analysis (PCoA) for microbial beta diversities between rewilding and control group. 17](#_Toc220921213)

[Figure S2. Correlation between salivary IL-6 and A) total salivary microbial richness, B) richness of functional gene pathways in saliva, and C) salivary gammaproteobacterial and D) alphaproteobacterial richness. 18](#_Toc220921214)

[References 19](#_Toc220921215)

# **Supplementary methods**

## Questionnaire data

Study questionnaire data were collected and managed using REDCap electronic data capture tools hosted at Tampere University ^1,2^. The study subjects filled out two electronic questionnaires of which the first one was a detailed background information questionnaire, including questions about the frequency of nature visits, medication use, diet, alcohol consumption, pets, and soil contact. Data for diet included berries, fruits, vegetables, fish, whole wheat, meat, processed meat, junk food, sweet confectionery, soft drinks, alcohol consumption, sweets, cereals, own garden, and nature products. Questionnaire was filled with scale: 0 = never, 1 = rarely, 2 = 1-4 times in a month, 3 = 2-6 times a week, 4 = daily.

The second questionnaire was about the yard management practices, and details that they do in the yard. They answered to the questionnaire at the baseline and after three-month study period. Yard management practices and activities in the yard were as following: Time spent at the yard, berry and fruit picking, vegetable, herbs and salad picking, renovation, chopping trees, vehicle maintenance, grilling and eating, nature observation, relaxation, sports, social gathering, planting, weeding, cutting plants, lawn mowing, irrigation, raking, mowing, insecticide, plant pesticide use, organic fertilizers use, inorganic fertilizers use, liming, and composting garden residuals. Questionnaire was filled with scale: 1 = rarely or never, 2 = 1-3 times in a month, 3 = once a week, 4 = many times in a week, 5 = daily and 6 = many times in a day, except house plants are the number of the plants indoors.

## Sample size calculation for study participants

Sample size calculations were performed to determine the required number of human study participants. The calculation was based on prior effect estimates from the studies indicating that the exposure to biodiversity modulates skin gammaproteobacterial diversity, which in turn is associated with immune regulation i.e. cytokine levels.^3–6^ Based on these studies, study subjects in contact with biodiversity (rewilding group) have an average of 17 genera of Gammaproteobacteria on their skin, while the control group has an average of 8 genera. Considering that the study includes three cities (clusters) and the coefficient of variation between clusters is 0.2 and when the significance level is set at p ≤ 0.05 and the statistical power is 0.8 (80.1%), the required sample size in each group per city is 5 subjects. These means 15 study subjects per treatment and altogether 30 participants. As in one of our previous studies one third of study subjects cancelled the participation, the target was to recruit 21 study subjects for each treatment group. The number of study subjects per cluster was calculated with *clusterPower* package using *cpa.count* function in R. This function implements the approach of Hayes and Bennett (1999).^8^

# Supplementary results

## Table S1. T test results for yard characteristic between control and rewilding groups at baseline.

Data is presented as mean ± standard deviation (sd). T teststatistics are reported as t statistics, p value, and Benjamini-Hochberg adjusted p value.

|  | Control | | Rewilding | | T test statistics | | |
| --- | --- | --- | --- | --- | --- | --- | --- |
|  | **Mean** | **sd** | **Mean** | **sd** | **t statistic** | **p value** | **adjusted p value** |
| Manicured% | 96 | 9 | 80 | 26 | 2.586 | **0.016** | **0.048** |
| Unkempt% | 4 | 9 | 20 | 26 | -2.586 | **0.016** | **0.048** |
| Whole yard area m2 | 559 | 367 | 581 | 468 | -0.157 | 0.876 | 1.000 |
| Gravel/sand% | 7 | 7 | 15 | 16 | -2.084 | **0.046** | 0.138 |
| Paved surfaces% | 18 | 13 | 14 | 14 | 0.877 | 0.387 | 1.000 |
| Lawn% | 45 | 14 | 37 | 22 | 1.192 | 0.241 | 0.724 |
| Meadow% | 0 | 0 | 8 | 17 | -2.030 | 0.056 | 0.168 |
| Tree canopy% | 13 | 10 | 19 | 20 | -1.272 | 0.213 | 0.638 |
| Flowers or other perennials% | 5 | 6 | 8 | 7 | -0.982 | 0.333 | 0.999 |
| Ornamental schrubs% | 16 | 8 | 9 | 9 | 2.495 | **0.018** | 0.054 |
| Berry bushes% | 1 | 2 | 6 | 8 | -2.327 | **0.029** | 0.088 |
| Rock% | 1 | 1 | 3 | 6 | -2.019 | 0.055 | 0.166 |
| Rock garden% | 0 | 0 | 1 | 3 | -1.278 | 0.215 | 0.646 |
| Fruit trees% | 4 | 5 | 7 | 12 | -1.355 | 0.186 | 0.557 |
| Bare organic ground% | 3 | 6 | 4 | 8 | -0.424 | 0.675 | 1.000 |
| Moss estimation of frequency | 7 | 6 | 15 | 19 | -1.760 | 0.090 | 0.271 |
| Plant richness summer | 85 | 25 | 82 | 30 | 0.341 | 0.736 | 1.000 |
| Plant richness fall | 89 | 25 | 100 | 29 | 1.090 | 0.287 | 0.600 |

## Table S2. Generalized linear mixed model (GLMM) results for yard activities and garden management practices, animal contacts, and nature visits A) between treatments on day 90 (fall), B) within rewilding and C) control groups between baseline (summer) and day 90 (fall).

Data is presented at ordinal scale (median ± confidence level 95% = cl): 1 = rarely or never, 2 = 1-3 times in a month, 3 = once a week, 4 = many times in a week, 5 = daily and 6 = many times in a day, except house plants are presented as mean ± standard deviation (sd). GLMM statistics are reported as z value, p value, Benjamini-Hochberg adjusted p value and fixed R squared (R^2^) for the model.

| A) Between treatments | Rewilding | | Control | | GLMM | | | |
| --- | --- | --- | --- | --- | --- | --- | --- | --- |
|  | **Median** | **cl** | **Median** | **cl** | **z value** | **p value** | **p adjust** | **R^2^** |
| Spending time at the yard | 4.32 | 1.11 | 4.50 | 0.93 | -0.43 | 0.670 | 0.914 | 0.01 |
| Berry and fruit picking | 1.89 | 1.24 | 2.00 | 1.60 | -0.19 | 0.848 | 0.914 | 0.00 |
| Vegetable picking | 1.16 | 0.37 | 1.38 | 0.74 | -0.99 | 0.321 | 0.886 | 0.04 |
| Herbs and salad picking | 1.42 | 0.77 | 1.63 | 1.06 | -0.57 | 0.567 | 0.886 | 0.01 |
| Renovation | 1.74 | 0.73 | 1.88 | 1.13 | -0.39 | 0.694 | 0.914 | 0.01 |
| Chopping trees | 1.32 | 0.67 | 1.50 | 1.07 | -0.56 | 0.579 | 0.886 | 0.01 |
| Vehicle maintenance | 1.05 | 0.23 | 1.13 | 0.35 | -0.64 | 0.524 | 0.886 | 0.02 |
| Grilling and eating | 1.26 | 0.56 | 1.13 | 0.35 | 0.64 | 0.521 | 0.886 | 0.02 |
| Nature observation | 3.47 | 1.26 | 3.38 | 1.77 | 0.17 | 0.864 | 0.914 | 0.00 |
| Relaxation | 2.21 | 1.47 | 2.13 | 1.13 | 0.15 | 0.879 | 0.914 | 0.00 |
| Sports | 1.32 | 0.82 | 1.25 | 0.71 | 0.20 | 0.838 | 0.914 | 0.00 |
| Social gathering | 2.21 | 1.27 | 2.63 | 1.19 | -0.80 | 0.424 | 0.886 | 0.02 |
| Planting | 1.84 | 0.76 | 1.13 | 0.35 | 1.99 | **0.002** | **0.052** | 0.22 |
| Weeding | 1.42 | 0.77 | 1.75 | 1.04 | -0.91 | 0.364 | 0.886 | 0.03 |
| Cutting plants | 1.63 | 0.76 | 2.13 | 0.99 | -1.35 | 0.177 | 0.886 | 0.07 |
| Lawn mowing | 1.21 | 0.42 | 1.25 | 0.46 | -0.23 | 0.822 | 0.914 | 0.00 |
| Irrigation | 1.89 | 0.99 | 1.63 | 1.06 | 0.65 | 0.519 | 0.886 | 0.02 |
| Raking | 2.00 | 1.20 | 2.88 | 1.13 | -1.66 | 0.097 | 0.841 | 0.10 |
| Composting garden residuals | 1.89 | 1.10 | 2.00 | 1.07 | -0.24 | 0.812 | 0.886 | 0.00 |
| House plants | 17.37 | 11.35 | 13.75 | 10.94 | 0.77 | 0.438 | 0.886 | 0.02 |
| Dog contact | 3.16 | 1.21 | 2.13 | 0.35 | 1.70 | 0.090 | 0.914 | 0.19 |
| Cat contact | 2.84 | 1.34 | 2.38 | 1.06 | 0.87 | 0.386 | 0.886 | 0.03 |
| Soil contact | 4.16 | 0.96 | 4.63 | 0.52 | -1.24 | 0.216 | 0.841 | 0.06 |
| Nature visits | 3.43 | 0.98 | 4.07 | 0.96 | -1.84 | 0.066 | 0.886 | 0.10 |
| B) Rewilding | **Baseline, summer** | | **Day 90, fall** | | **GLMM** | | | |
|  | **Median** | **cl** | **Median** | **cl** | **z value** | **p value** | **P adjust** | **R^2^** |
| Spending time at the yard | 5.33 | 0.80 | 4.32 | 1.11 | -2.75 | **0.006** | **0.022** | 0.23 |
| Berry and fruit picking | 1.76 | 1.04 | 1.89 | 1.24 | 0.38 | 0.707 | 0.875 | 0.00 |
| Vegetable picking | 1.29 | 0.64 | 1.16 | 0.37 | -0.76 | 0.450 | 0.650 | 0.01 |
| Herbs and salad picking | 2.57 | 1.25 | 1.42 | 0.77 | -2.75 | **0.006** | **0.022** | 0.24 |
| Renovation | 2.57 | 1.43 | 1.74 | 0.73 | -2.04 | **0.041** | 0.107 | 0.12 |
| Chopping trees | 1.05 | 0.22 | 1.32 | 0.67 | 1.45 | 0.148 | 0.257 | 0.07 |
| Vehicle maintenance | 1.24 | 0.44 | 1.05 | 0.23 | -1.50 | 0.132 | 0.245 | 0.07 |
| Grilling and eating | 2.90 | 1.22 | 1.26 | 0.56 | -3.11 | **0.002** | **0.010** | 0.46 |
| Nature observation | 4.62 | 1.28 | 3.47 | 1.26 | -2.47 | **0.013** | **0.042** | 0.17 |
| Relaxation | 4.24 | 1.41 | 2.21 | 1.47 | -3.28 | **0.001** | **0.009** | 0.36 |
| Sports | 1.67 | 1.06 | 1.32 | 0.82 | -1.13 | 0.259 | 0.401 | 0.03 |
| Social gathering | 4.10 | 1.30 | 2.21 | 1.27 | -3.31 | **0.001** | **0.009** | 0.35 |
| Planting | 2.43 | 1.43 | 1.84 | 0.76 | -1.51 | 0.131 | 0.245 | 0.06 |
| Weeding | 3.05 | 1.24 | 1.42 | 0.77 | -3.10 | **0.002** | **0.010** | 0.43 |
| Cutting plants | 2.33 | 1.28 | 1.63 | 0.76 | -1.92 | 0.055 | 0.130 | 0.10 |
| Lawn mowing | 1.62 | 0.67 | 1.21 | 0.42 | -2.08 | **0.037** | 0.107 | 0.12 |
| Irrigation | 3.90 | 1.51 | 1.89 | 0.99 | -3.39 | **0.001** | **0.009** | 0.39 |
| Raking | 1.62 | 0.92 | 2.00 | 1.20 | 1.12 | 0.262 | 0.401 | 0.03 |
| Composting garden residuals | 2.14 | 1.28 | 1.89 | 1.10 | -0.67 | 0.505 | 0.691 | 0.01 |
| House plants | 18.81 | 12.93 | 17.37 | 11.35 | -0.38 | 0.703 | 0.875 | 0.00 |
| Dog contact | 3.14 | 1.15 | 3.16 | 1.21 | 0.04 | 0.967 | 0.967 | 0.00 |
| Cat contact | 2.76 | 1.30 | 2.84 | 1.34 | 0.20 | 0.844 | 0.900 | 0.00 |
| Soil contact | 4.24 | 0.94 | 4.16 | 0.96 | -0.27 | 0.785 | 0.900 | 0.00 |
| Nature visits | 3.43 | 0.98 | 3.37 | 0.96 | -0.20 | 0.840 | 0.900 | 0.00 |
| C) Control group | **Baseline, summer** | | **Day 90, fall** | | **GLMM** | | | |
|  | **Median** | **cl** | **Median** | **cl** | **z value** | **p value** | **P adjust** | **R^2^** |
| Spending time at the yard | 5.53 | 0.64 | 4.50 | 0.93 | -2.29 | **0.022** | 0.143 | 0.32 |
| Berry and fruit picking | 2.27 | 1.67 | 2.00 | 1.60 | -0.38 | 0.700 | 0.827 | 0.01 |
| Vegetable picking | 1.73 | 1.53 | 1.38 | 0.74 | -0.63 | 0.531 | 0.727 | 0.02 |
| Herbs and salad picking | 2.80 | 1.74 | 1.63 | 1.06 | -1.58 | 0.113 | 0.294 | 0.12 |
| Renovation | 1.87 | 0.92 | 1.88 | 1.13 | 0.02 | 0.984 | 0.984 | 0.00 |
| Chopping trees | 1.07 | 0.26 | 1.50 | 1.07 | 1.15 | 0.250 | 0.542 | 0.10 |
| Vehicle maintenance | 1.40 | 0.63 | 1.13 | 0.35 | -1.08 | 0.280 | 0.560 | 0.06 |
| Grilling and eating | 2.80 | 1.01 | 1.13 | 0.35 | -2.34 | **0.019** | 0.143 | 0.56 |
| Nature observation | 4.00 | 1.85 | 3.38 | 1.77 | -0.80 | 0.424 | 0.648 | 0.03 |
| Relaxation | 3.73 | 1.71 | 2.13 | 1.13 | -2.04 | **0.042** | 0.156 | 0.20 |
| Sports | 1.53 | 0.74 | 1.25 | 0.71 | -0.88 | 0.378 | 0.648 | 0.04 |
| Social gathering | 3.87 | 1.55 | 2.63 | 1.19 | -1.77 | 0.077 | 0.222 | 0.15 |
| Planting | 1.80 | 0.77 | 1.13 | 0.35 | -1.87 | 0.062 | 0.202 | 0.22 |
| Weeding | 2.80 | 0.86 | 1.75 | 1.04 | -2.12 | **0.034** | 0.147 | 0.29 |
| Cutting plants | 2.67 | 0.90 | 2.13 | 0.99 | -1.29 | 0.198 | 0.468 | 0.08 |
| Lawn mowing | 2.13 | 0.83 | 1.25 | 0.46 | -2.16 | **0.031** | 0.147 | 0.26 |
| Irrigation | 3.40 | 1.30 | 1.63 | 1.06 | -2.44 | **0.015** | 0.143 | 0.35 |
| Raking | 1.67 | 0.72 | 2.88 | 1.13 | 2.31 | **0.021** | 0.143 | 0.33 |
| Composting garden residuals | 2.07 | 1.33 | 2.00 | 1.07 | -0.13 | 0.899 | 0.935 | 0.00 |
| House plants | 15.33 | 11.41 | 13.75 | 10.94 | -0.33 | 0.738 | 0.834 | 0.00 |
| Dog contact | 2.40 | 0.83 | 2.13 | 0.35 | -0.84 | 0.401 | 0.648 | 0.04 |
| Cat contact | 2.20 | 0.77 | 2.38 | 1.06 | 0.47 | 0.641 | 0.796 | 0.01 |
| Soil contact | 4.40 | 0.63 | 4.63 | 0.52 | 0.87 | 0.382 | 0.648 | 0.03 |
| Nature visits | 4.07 | 0.96 | 4.25 | 0.89 | 0.46 | 0.643 | 0.796 | 0.01 |

## **Table S3.** **Linear mixed model (LMM) results for correlation between skin microbiota on the forearm and polypore species richness and decayed deadwood at A) baseline (summer) and B) day 90 (fall).**

Two separate models were run, one for deadwood and one for polypore species richness. Data is presented as mean ± standard deviation (sd). LMM statistics are reported as t value, p value, Benjamini-Hochberg adjusted p value and fixed R squared (R^2^) for the model.

| **A) Baseline (summer)** | **Rewilding** | | **Control** | | **LMM: Polypore species richness** | | | | **LMM: Decayed deadwood** | | | |
| --- | --- | --- | --- | --- | --- | --- | --- | --- | --- | --- | --- | --- |
|  | **Mean** | **sd** | **Mean** | **sd** | **t value** | **p value** | **p adjust** | **R^2^** | **t value** | **p value** | **p adjust** | **R^2^** |
| Order Burkholderiales | 2.49 | 3.08 | 2.06 | 2.62 | 3.90 | **0.0001** | **0.0002** | 0.82 | 2.83 | **0.0046** | **0.0092** | 0.58 |
| Family Burkholderiaceae | 0.23 | 0.47 | 0.24 | 0.54 | 2.83 | **0.0047** | **0.0047** | 0.78 | 2.85 | **0.0044** | **0.0092** | 0.57 |
| Family Neisseriaceae | 1.74 | 2.71 | 1.44 | 1.92 | 3.90 | **0.0001** | **0.0002** | 0.82 | 2.26 | **0.0237** | **0.0356** | 0.54 |
| Genus Eikenella | 0.11 | 0.42 | 0.10 | 0.36 | 4.40 | **0.0000** | **0.0001** | 0.84 | 3.66 | **0.0003** | **0.0015** | 0.64 |
| Family Paludibacteraceae | 0.01 | 0.03 | 0.01 | 0.02 | 2.92 | **0.0035** | **0.0042** | 0.79 | 1.95 | 0.0512 | 0.0512 | 0.52 |
| Genus F0058 | 0.01 | 0.03 | 0.01 | 0.02 | 3.12 | **0.0018** | **0.0027** | 0.80 | 2.07 | **0.0385** | **0.0463** | 0.53 |
| **B) Day 90 (fall)** | **Rewilding** | | **Control** | | **LMM: Polypore species richness** | | | | **LMM: Decayed deadwood** | | | |
|  | **Mean** | **sd** | **Mean** | **sd** | **t value** | **p value** | **p adjust** | **R^2^** | **t value** | **p value** | **p adjust** | **R^2^** |
| Order Burkholderiales | 2.90 | 3.51 | 1.54 | 1.84 | 1.82 | 0.0695 | 0.1042 | 0.74 | 0.54 | 0.5884 | 0.7061 | 0.42 |
| Family Burkholderiaceae | 0.18 | 0.41 | 0.10 | 0.12 | 0.90 | 0.3690 | 0.3690 | 0.72 | 1.13 | 0.2597 | 0.3895 | 0.44 |
| Family Neisseriaceae | 2.37 | 3.40 | 1.25 | 1.83 | 1.55 | 0.1201 | 0.1441 | 0.74 | 0.31 | 0.7530 | 0.7530 | 0.42 |
| Genus Eikenella | 0.05 | 0.18 | 0.05 | 0.10 | 7.18 | **0.0000** | **0.0000** | 0.90 | 3.77 | **0.0002** | **0.0010** | 0.63 |
| Family Paludibacteraceae | 0.02 | 0.08 | 0.004 | 0.01 | 4.82 | **0.0000** | **0.0000** | 0.84 | 3.37 | **0.0007** | **0.0015** | 0.59 |
| Genus F0058 | 0.02 | 0.08 | 0.004 | 0.01 | 4.82 | **0.0000** | **0.0000** | 0.84 | 3.37 | **0.0007** | **0.0015** | 0.59 |

## Table S4. Correlation between beta diversity of skin bacteria on A) back of the hand and B) forearm, and decaying deadwood amount (N) and polypores at baseline and day 90. Beta diversity is reported for total bacterial community level (ASV).

Correlation was assessed using function envfit function in R and statistics are reported as the square of the correlation (R^2^), p value, and Benjamin-Hochberg adjusted p value. Significance is tested by permutation test (999 permutations).

| A) Back of the hand | Baseline | | | Day 90 | | |
| --- | --- | --- | --- | --- | --- | --- |
|  | **R^2^** | **P value** | **P adjust** | **R^2^** | **P value** | **P adjust** |
| N deadwood all | 0.083 | 0.226 | 0.483 | 0.150 | 0.089 | 0.099 |
| N deadwood decayed | 0.083 | 0.226 | 0.483 | 0.298 | **0.035** | 0.090 |
| N deadwood decayed deciduous | 0.063 | 0.338 | 0.483 | 0.180 | **0.047** | 0.090 |
| N polypores deadwood | 0.067 | 0.297 | 0.483 | 0.183 | 0.058 | 0.090 |
| N polypores total | 0.071 | 0.275 | 0.483 | 0.193 | **0.042** | 0.090 |
| Polypore species richness | 0.064 | 0.328 | 0.483 | 0.242 | **0.017** | 0.090 |
| Datronia mollis | 0.008 | 0.877 | 0.938 | 0.216 | **0.028** | 0.090 |
| Fomes fomentarius | 0.043 | 0.491 | 0.614 | 0.159 | 0.072 | 0.090 |
| Phellinus igniarius | 0.004 | 0.938 | 0.938 | 0.052 | 0.496 | 0.496 |
| Phellinus laevigatus | 0.074 | 0.278 | 0.483 | 0.177 | **0.049** | 0.090 |
| B) Forearm | **Baseline** | | | **Day 90** | | |
|  | **R^2^** | **P value** | **P adjust** | **R^2^** | **P value** | **P adjust** |
| N deadwood all | 0.175 | **0.043** | 0.059 | 0.045 | 0.523 | 0.981 |
| N deadwood decayed | 0.215 | **0.024** | 0.059 | 0.029 | 0.674 | 0.981 |
| N deadwood decayed deciduous | 0.199 | **0.029** | 0.059 | 0.006 | 0.940 | 0.994 |
| N polypores deadwood | 0.172 | 0.053 | 0.059 | 0.026 | 0.719 | 0.981 |
| N polypores total | 0.175 | **0.046** | 0.059 | 0.023 | 0.734 | 0.981 |
| Polypore species richness | 0.189 | **0.037** | 0.059 | 0.052 | 0.453 | 0.981 |
| Datronia mollis | 0.028 | 0.655 | 0.655 | 0.045 | 0.514 | 0.981 |
| Fomes fomentarius | 0.195 | **0.037** | 0.059 | 0.001 | 0.994 | 0.994 |
| Phellinus igniarius | 0.180 | **0.038** | 0.059 | 0.025 | 0.714 | 0.981 |
| Phellinus laevigatus | 0.181 | 0.052 | 0.059 | 0.018 | 0.785 | 0.981 |

## Table S5. ANCOM-BC2 results for the association between neighborhood deadwood abundance and (A) oral microbiota and (B) oral functional gene pathways.

Effect estimates are reported as log fold change for log10-transformed neighborhood deadwood abundance (LFC), along with standard error (SE), Wald p-value, and Benjamini–Hochberg adjusted p-value (q-value). Taxa shown passed the sample-size screening criterion (passed_ss = TRUE).

| A) Oral microbiota | Baseline | | | | Day 90 | | | |
| --- | --- | --- | --- | --- | --- | --- | --- | --- |
|  | **LFC** | **SE** | **p value** | **q value** | **LFC** | **SE** | **p value** | **q value** |
| Brachybacterium vulturis | 0.22 | 0.06 | **0.005** | **0.050** | 0.18 | 0.05 | **0.010** | 0.069 |
| Corynebacterium segmentosum | -0.34 | 0.06 | **0.005** | **0.050** | -0.21 | 0.06 | **0.011** | 0.070 |
| Streptomyces sp. HSG2 | 0.32 | 0.06 | **0.005** | 0.051 | 0.23 | 0.05 | **0.005** | **0.045** |
| Leucobacter insecticola | -0.23 | 0.06 | **0.008** | 0.060 | -0.24 | 0.06 | **0.006** | **0.050** |
| Cytobacillus sp. CY-G | 0.90 | 0.06 | **0.0001** | **0.008** | 0.31 | 0.06 | **0.006** | **0.050** |
| B) Oral gene pathways | **Baseline** | | | | **Day 90** | | | |
|  | **LFC** | **SE** | **p value** | **q value** | **LFC** | **SE** | **p value** | **q value** |
| Superpathway of fatty acids biosynthesis, E. coli | 0.37 | 0.07 | **0.004** | **0.048** | -0.12 | 0.06 | 0.093 | 0.277 |
| Tetrapyrrole biosynthesis II from glycine, unclassified | -0.25 | 0.07 | **0.005** | **0.050** | -0.14 | 0.08 | 0.101 | 0.297 |
| Superpathway of L phenylalanine biosynthesis | 0.28 | 0.09 | **0.005** | **0.050** | -0.07 | 0.07 | 0.349 | 0.635 |
| C4 photosynthetic carbon assimilation cycle,  NAD ME type unclassified | -0.30 | 0.09 | **0.005** | **0.050** | 0.13 | 0.07 | 0.110 | 0.310 |
| ppGpp metabolism, unclassified | -0.27 | 0.08 | **0.005** | 0.052 | 0.03 | 0.09 | 0.742 | 0.921 |

## **Table S6. Age, gender, body mass index (BMI), pet ownership, and diet association with A) salivary microbial species, and B) functional gene pathways, and C) skin bacteria on the back of the hand, and D) forearm, and cytokines E) IL-10 and F) IL-6.).**

Data for diet was analyzed at ordinal scale (median ± confidence level 95% = cl): 0 = never, 1 = rarely, 2 = 1-4 times in a month, 3 = 2-6 times a week, 4 = daily. Salivary and skin microbial associations were analyzed with PERMANOVA, and statistics are reported as R squared (R^2^), F value and p value. GLM statistics for cytokines are reported as standard error, t value, and p value.

| A) Salivary microbial species | R^2^ | F value | P value |  | B) Salivary functional gene pathways | R^2^ | F value | P value |  | |
| --- | --- | --- | --- | --- | --- | --- | --- | --- | --- | --- |
| Age | 0.03 | 0.93 | 0.518 |  | Age | 0.00 | 0.27 | 0.741 |  | |
| Gender | 0.02 | 0.67 | 0.944 |  | Gender | 0.12 | 7.62 | 0.093 |  | |
| BMI | 0.03 | 0.72 | 0.857 |  | BMI | 0.01 | 0.65 | 0.473 |  | |
| Pet ownership | 0.04 | 0.96 | 0.509 |  | Pet ownership | 0.08 | 4.50 | 0.150 |  | |
| Diet: |  |  |  |  | Diet: |  |  |  |  | |
| Berries | 0.13 | 1.16 | 0.213 |  | Berries | 0.07 | 1.50 | 0.353 |  | |
| Fruits | 0.10 | 0.92 | 0.619 |  | Fruits | 0.19 | 4.12 | 0.187 |  | |
| Vegetables | 0.02 | 0.64 | 0.946 |  | Vegetables | 0.06 | 4.11 | 0.091 |  | |
| Fish | 0.05 | 0.66 | 0.968 |  | Fish | 0.09 | 2.78 | 0.215 |  | |
| Wholewheat | 0.07 | 1.00 | 0.466 |  | Wholewheat | 0.03 | 0.87 | 0.407 |  | |
| Meat | 0.12 | 0.86 | 0.772 |  | Meat | 0.08 | 1.23 | 0.365 |  | |
| Processed meat | 0.12 | 0.82 | 0.841 |  | Processed meat | 0.20 | 3.26 | 0.181 |  | |
| Junk food | 0.06 | 0.86 | 0.698 |  | Junk food | 0.01 | 0.25 | 0.836 |  | |
| Sweet confectionery | 0.09 | 0.85 | 0.780 |  | Sweet confectionery | 0.08 | 1.82 | 0.292 |  | |
| Soft drinks | 0.13 | 0.97 | 0.531 |  | Soft drinks | 0.07 | 0.39 | 0.812 |  | |
| Alcohol consumption | 0.14 | 1.06 | 0.353 |  | Alcohol consumption | 0.04 | 0.20 | 0.934 |  | |
| Sweets | 0.12 | 0.91 | 0.715 |  | Sweets | 0.22 | 1.26 | 0.265 |  | |
| Cereals | 0.12 | 0.94 | 0.664 |  | Cereals | 0.02 | 0.10 | 0.929 |  | |
| Own garden or nature products | 0.04 | 1.21 | 0.193 |  | Own garden or nature products | 0.06 | 1.43 | 0.228 |  | |
| C) Skin bacteria, back of the hand | **R^2^** | **F value** | **P value** | **Padj** | **D) Skin bacteria, forearm** | **R^2^** | **F value** | **P value** | **Padj** | |
| Age | 0.02 | 1.00 | 0.430 | 0.49 | Age | 0.04 | 1.59 | **0.037** | | 0.33 |
| Gender | 0.03 | 1.46 | 0.079 | 0.27 | Gender | 0.03 | 1.15 | 0.267 | 0.43 | |
| BMI | 0.02 | 0.93 | 0.525 | 0.53 | BMI | 0.02 | 0.94 | 0.552 | 0.78 | |
| Pet ownership | 0.03 | 1.11 | 0.313 | 0.49 | Pet ownership | 0.03 | 1.15 | 0.280 | 0.43 | |
| Diet: |  |  |  |  | Diet: |  |  |  | 0.43 | |
| Berries | 0.11 | 1.28 | 0.120 | 0.34 | Berries | 0.12 | 1.17 | 0.215 | 0.43 | |
| Fruits | 0.08 | 1.23 | 0.172 | 0.37 | Fruits | 0.10 | 1.30 | 0.108 | 0.43 | |
| Vegetables | 0.05 | 1.15 | 0.282 | 0.49 | Vegetables | 0.06 | 1.26 | 0.153 | 0.33 | |
| Fish | 0.10 | 1.51 | **0.043** | 0.18 | Fish | 0.11 | 1.42 | **0.045** | 0.43 | |
| Wholewheat | 0.04 | 0.96 | 0.508 | 0.53 | Wholewheat | 0.06 | 1.23 | 0.183 | 0.43 | |
| Meat | 0.11 | 1.26 | 0.143 | 0.35 | Meat | 0.11 | 1.13 | 0.246 | 0.43 | |
| Processed meat | 0.13 | 1.42 | **0.022** | 0.12 | Processed meat | 0.12 | 1.21 | 0.150 | 0.91 | |
| Junk food | 0.08 | 1.86 | **0.004** | 0.07 | Junk food | 0.04 | 0.82 | 0.843 | 0.33 | |
| Sweet confectionery | 0.11 | 1.59 | **0.012** | 0.10 | Sweet confectionery | 0.10 | 1.35 | 0.058 | 0.91 | |
| Soft drinks | 0.11 | 0.99 | 0.430 | 0.49 | Soft drinks | 0.11 | 0.87 | 0.804 | 0.91 | |
| Sweets | 0.12 | 1.04 | 0.370 | 0.49 | Sweets | 0.11 | 0.84 | 0.855 | 0.98 | |
| Cereals | 0.12 | 1.09 | 0.321 | 0.49 | Cereals | 0.09 | 0.72 | 0.982 | 0.91 | |
| Own garden or nature products | 0.06 | 1.03 | 0.427 | 0.49 | Own garden or nature products | 0.05 | 0.84 | 0.828 |  | |
|  |  |  |  |  |  |  |  |  |  | |
| E) IL-10 | **Standard error** | **t value** | **P value** |  | **F) IL-6** | **Standard error** | **t value** | **P value** |  | |
| Age | 0.95 | -0.12 | 0.906 |  | Age | 0.12 | -1.35 | 0.200 |  | |
| Gender | 19.51 | -0.15 | 0.887 |  | Gender | 2.03 | -1.60 | 0.135 |  | |
| BMI | 4.23 | 0.14 | 0.889 |  | BMI | 0.50 | 0.76 | 0.462 |  | |
| Pet ownership | 17.61 | -0.03 | 0.976 |  | Pet ownership | 3.12 | 0.32 | 0.759 |  | |
| Diet: |  |  |  |  | Diet: |  |  |  |  | |
| Berries | 15.77 | -0.06 | 0.953 |  | Berries | 1.18 | -0.82 | 0.430 |  | |
| Fruits | 41.56 | 0.16 | 0.876 |  | Fruits | 1.47 | 1.06 | 0.310 |  | |
| Fish | 42.69 | -0.10 | 0.922 |  | Fish | 1.62 | -0.44 | 0.669 |  | |
| Wholewheat | 11.31 | -0.01 | 0.995 |  | Wholewheat | 5.01 | 0.56 | 0.588 |  | |
| Vegetables | 76.95 | 0.16 | 0.874 |  | Vegetables | 4.29 | 1.10 | 0.291 |  | |
| Meat | 17.17 | -0.02 | 0.983 |  | Meat | 1.42 | -0.60 | 0.562 |  | |
| Processed meat | 21.93 | -0.16 | 0.875 |  | Processed meat | 2.31 | -0.81 | 0.436 |  | |
| Junk food | 25.28 | 0.02 | 0.986 |  | Junk food | 3.14 | 0.57 | 0.581 |  | |
| Sweet confectionery | 39.41 | -0.18 | 0.859 |  | Sweet confectionery | 3.76 | -0.92 | 0.376 |  | |
| Soft drinks | 8.07 | 0.08 | 0.939 |  | Soft drinks | 1.04 | 0.17 | 0.864 |  | |
| Sweets | 16.49 | 0.11 | 0.917 |  | Sweets | 0.93 | -0.05 | 0.960 |  | |
| Cereals | 18.02 | 0.16 | 0.873 |  | Cereals | 2.19 | 1.03 | 0.322 |  | |
| Own garden or nature products | 47328.11 | 0.00 | 0.999 |  | Own garden or nature products | 5.83 | 0.62 | 0.545 |  | |
| Alcohol consumption | 25.30 | -0.17 | 0.864 |  | Alcohol consumption | 2.12 | -1.63 | 0.129 |  | |

## Table S7. Linear mixed model (LMM) results between baseline and fall in skin bacterial richness and diversity on the back of the hand within A) rewilding and B) control treatment, and with C) all study subjects in the model, and on the forearm within D) rewilding and E) control treatment, and with F) all study subjects in the model.

Data is presented as mean ± standard deviation (sd). LMM statistics are reported as t value, p value, Benjamini-Hochberg adjusted p value and R squared (R^2^) for the model.

| A) Back of the hand, rewilding group | Baseline | | | | Fall | | | | LMM | | | | | | |
| --- | --- | --- | --- | --- | --- | --- | --- | --- | --- | --- | --- | --- | --- | --- | --- |
|  | **Mean** | | **sd** | | **Mean** | | **sd** | | **t value** | | **p value** | | **p adjust** | | **R^2^** |
| Total richness | 248.76 | | 82.83 | | 228.81 | | 66.51 | | -1.07 | | 0.286 | | 0.531 | | 0.02 |
| Phyla Proteobacteria richness | 50.62 | | 30.41 | | 43.19 | | 26.46 | | -0.84 | | 0.398 | | 0.531 | | 0.02 |
| Class Gammaproteobacteria richness | 29.76 | | 15.26 | | 27.76 | | 17.94 | | -0.39 | | 0.697 | | 0.711 | | 0.00 |
| Class Alphaproteobacteria richness | 20.71 | | 17.85 | | 15.33 | | 9.83 | | -1.21 | | 0.226 | | 0.531 | | 0.04 |
| Total shannon | 4.98 | | 0.36 | | 4.90 | | 0.40 | | -1.00 | | 0.317 | | 0.531 | | 0.01 |
| Phyla Proteobacteria shannon | 3.31 | | 0.52 | | 3.12 | | 0.58 | | -1.14 | | 0.254 | | 0.531 | | 0.03 |
| Class Gammaproteobacteria shannon | 2.95 | | 0.54 | | 2.81 | | 0.62 | | -0.86 | | 0.387 | | 0.531 | | 0.02 |
| Class Alphaproteobacteria shannon | 2.27 | | 0.58 | | 2.11 | | 0.51 | | -1.03 | | 0.301 | | 0.531 | | 0.02 |
| Total simpson | 0.99 | | 0.01 | | 0.99 | | 0.01 | | -0.37 | | 0.711 | | 0.711 | | 0.00 |
| Phyla Proteobacteria simpson | 0.95 | | 0.02 | | 0.93 | | 0.04 | | -1.90 | | 0.057 | | 0.531 | | 0.07 |
| Class Gammaproteobacteria simpson | 0.93 | | 0.04 | | 0.91 | | 0.05 | | -1.47 | | 0.142 | | 0.531 | | 0.03 |
| Class Alphaproteobacteria simpson | 0.84 | | 0.08 | | 0.83 | | 0.08 | | -0.69 | | 0.488 | | 0.586 | | 0.01 |
| B) Back of the hand, control group | **Baseline** | | | | **Fall** | | | | **LMM** | | | | | | |
|  | **Mean** | | **sd** | | **Mean** | | **sd** | | **t value** | | **p value** | | **p adjust** | | **R^2^** |
| Total richness | 250.57 | | 81.58 | | 213.00 | | 63.71 | | -1.25 | | 0.210 | | 0.248 | | 0.06 |
| Phyla Proteobacteria richness | 45.36 | | 24.24 | | 32.82 | | 14.20 | | -1.52 | | 0.129 | | 0.223 | | 0.09 |
| Class Gammaproteobacteria richness | 23.50 | | 14.69 | | 16.27 | | 6.48 | | -1.51 | | 0.130 | | 0.223 | | 0.09 |
| Class Alphaproteobacteria richness | 21.71 | | 12.57 | | 16.55 | | 10.32 | | -1.10 | | 0.271 | | 0.271 | | 0.05 |
| Total shannon | 4.90 | | 0.46 | | 4.69 | | 0.38 | | -1.21 | | 0.227 | | 0.248 | | 0.06 |
| Phyla Proteobacteria shannon | 3.13 | | 0.57 | | 2.75 | | 0.55 | | -1.70 | | 0.089 | | 0.214 | | 0.11 |
| Class Gammaproteobacteria shannon | 2.63 | | 0.53 | | 2.25 | | 0.51 | | -1.77 | | 0.077 | | 0.214 | | 0.12 |
| Class Alphaproteobacteria shannon | 2.39 | | 0.52 | | 2.09 | | 0.53 | | -1.38 | | 0.167 | | 0.245 | | 0.08 |
| Total simpson | 0.99 | | 0.00 | | 0.99 | | 0.01 | | -1.33 | | 0.184 | | 0.245 | | 0.06 |
| Phyla Proteobacteria simpson | 0.93 | | 0.04 | | 0.89 | | 0.08 | | -1.80 | | 0.071 | | 0.214 | | 0.12 |
| Class Gammaproteobacteria simpson | 0.90 | | 0.05 | | 0.84 | | 0.11 | | -1.82 | | 0.069 | | 0.214 | | 0.13 |
| Class Alphaproteobacteria simpson | 0.87 | | 0.06 | | 0.81 | | 0.07 | | -2.10 | | **0.036** | | 0.214 | | 0.15 |
| C) Back of the hand, all study subjects | **Baseline** | | | | **Fall** | | | | **LMM** | | | | | | |
|  | **Mean** | | **sd** | | **Mean** | | **sd** | | **t value** | | **p value** | | **p adjust** | | **R^2^** |
| Total richness | 245.67 | | 79.36 | | 226.82 | | 66.94 | | -1.31 | | 0.192 | | 0.263 | | 0.03 |
| Phyla Proteobacteria richness | 47.11 | | 27.45 | | 40.52 | | 23.47 | | -1.56 | | 0.119 | | 0.237 | | 0.06 |
| Class Gammaproteobacteria richness | 27.22 | | 14.92 | | 23.82 | | 15.63 | | -1.01 | | 0.313 | | 0.376 | | 0.06 |
| Class Alphaproteobacteria richness | 19.78 | | 15.31 | | 16.61 | | 10.87 | | -1.57 | | 0.116 | | 0.237 | | 0.07 |
| Total shannon | 4.92 | | 0.40 | | 4.85 | | 0.40 | | -0.92 | | 0.357 | | 0.390 | | 0.02 |
| Phyla Proteobacteria shannon | 3.21 | | 0.52 | | 3.02 | | 0.59 | | -1.90 | | 0.058 | | 0.174 | | 0.07 |
| Class Gammaproteobacteria shannon | 2.82 | | 0.54 | | 2.62 | | 0.63 | | -1.29 | | 0.197 | | 0.263 | | 0.07 |
| Class Alphaproteobacteria shannon | 2.26 | | 0.54 | | 2.14 | | 0.55 | | -2.33 | | **0.020** | | 0.118 | | 0.13 |
| Total simpson | 0.99 | | 0.01 | | 0.99 | | 0.01 | | -0.08 | | 0.937 | | 0.937 | | 0.03 |
| Phyla Proteobacteria simpson | 0.94 | | 0.03 | | 0.92 | | 0.06 | | -2.06 | | **0.039** | | 0.156 | | 0.08 |
| Class Gammaproteobacteria simpson | 0.92 | | 0.05 | | 0.89 | | 0.08 | | -1.42 | | 0.156 | | 0.263 | | 0.06 |
| Class Alphaproteobacteria simpson | 0.86 | | 0.07 | | 0.78 | | 0.08 | | -3.00 | | **0.003** | | **0.033** | | 0.15 |
| D) Forearm, rewilding group | **Baseline** | | | **Fall** | | | | **LMM** | | | | | | | |
|  | **Mean** | **sd** | | **Mean** | | **sd** | | **t value** | | **p value** | | **p adjust** | | **R^2^** | |
| Total richness | 230.76 | 93.03 | | 215.71 | | 77.66 | | -0.57 | | 0.566 | | 0.880 | | 0.01 | |
| Phyla Proteobacteria richness | 46.90 | 31.09 | | 40.00 | | 18.88 | | -0.87 | | 0.384 | | 0.880 | | 0.02 | |
| Class Gammaproteobacteria richness | 24.00 | 11.73 | | 25.52 | | 14.58 | | 0.38 | | 0.703 | | 0.880 | | 0.00 | |
| Class Alphaproteobacteria richness | 22.67 | 21.85 | | 14.33 | | 6.84 | | -1.67 | | 0.095 | | 0.382 | | 0.06 | |
| Total shannon | 4.87 | 0.37 | | 4.81 | | 0.61 | | -0.38 | | 0.707 | | 0.880 | | 0.00 | |
| Phyla Proteobacteria shannon | 3.09 | 0.63 | | 3.05 | | 0.62 | | -0.19 | | 0.850 | | 0.880 | | 0.00 | |
| Class Gammaproteobacteria shannon | 2.58 | 0.65 | | 2.69 | | 0.76 | | 0.47 | | 0.636 | | 0.880 | | 0.01 | |
| Class Alphaproteobacteria shannon | 2.40 | 0.63 | | 2.08 | | 0.39 | | -2.00 | | **0.045** | | 0.382 | | 0.09 | |
| Total simpson | 0.99 | 0.00 | | 0.98 | | 0.02 | | -0.98 | | 0.325 | | 0.880 | | 0.02 | |
| Phyla Proteobacteria simpson | 0.92 | 0.05 | | 0.92 | | 0.07 | | -0.18 | | 0.858 | | 0.880 | | 0.00 | |
| Class Gammaproteobacteria simpson | 0.88 | 0.10 | | 0.89 | | 0.10 | | 0.15 | | 0.880 | | 0.880 | | 0.00 | |
| Class Alphaproteobacteria simpson | 0.86 | 0.08 | | 0.82 | | 0.08 | | -1.67 | | 0.095 | | 0.382 | | 0.07 | |
| E) Forearm, control group | **Baseline** | | | **Fall** | | | | **LMM** | | | | | | | |
|  | **Mean** | **sd** | | **Mean** | | **sd** | | **t value** | | **p value** | | **p adjust** | | **R^2^** | |
| Total richness | 239.86 | 100.89 | | 199.67 | | 37.03 | | -1.56 | | 0.119 | | 0.159 | | 0.07 | |
| Phyla Proteobacteria richness | 44.86 | 29.78 | | 29.58 | | 16.82 | | -1.66 | | 0.097 | | 0.159 | | 0.09 | |
| Class Gammaproteobacteria richness | 21.14 | 12.96 | | 16.17 | | 13.14 | | -1.24 | | 0.214 | | 0.233 | | 0.04 | |
| Class Alphaproteobacteria richness | 23.64 | 22.75 | | 13.25 | | 8.79 | | -1.49 | | 0.137 | | 0.165 | | 0.08 | |
| Total shannon | 4.86 | 0.39 | | 4.70 | | 0.31 | | -1.89 | | 0.059 | | 0.142 | | 0.05 | |
| Phyla Proteobacteria shannon | 3.10 | 0.59 | | 2.66 | | 0.63 | | -2.15 | | **0.032** | | 0.096 | | 0.12 | |
| Class Gammaproteobacteria shannon | 2.49 | 0.65 | | 2.16 | | 0.69 | | -1.58 | | 0.113 | | 0.159 | | 0.06 | |
| Class Alphaproteobacteria shannon | 2.40 | 0.58 | | 1.89 | | 0.62 | | -2.33 | | **0.020** | | 0.089 | | 0.16 | |
| Total simpson | 0.99 | 0.00 | | 0.99 | | 0.00 | | -0.31 | | 0.758 | | 0.758 | | 0.00 | |
| Phyla Proteobacteria simpson | 0.93 | 0.04 | | 0.88 | | 0.07 | | -2.29 | | **0.022** | | 0.089 | | 0.16 | |
| Class Gammaproteobacteria simpson | 0.88 | 0.07 | | 0.82 | | 0.12 | | -1.57 | | 0.117 | | 0.159 | | 0.08 | |
| Class Alphaproteobacteria simpson | 0.86 | 0.07 | | 0.75 | | 0.16 | | -2.66 | | **0.008** | | 0.089 | | 0.19 | |
| F) Forearm, all study subjects | **Baseline** | | | **Fall** | | | | **LMM** | | | | | | | |
|  | **Mean** | **sd** | | **Mean** | | **sd** | | **t value** | | **p value** | | **p adjust** | | **R^2^** | |
| Total richness | 234.40 | 94.89 | | 209.88 | | 65.59 | | -1.23 | | 0.218 | | 0.291 | | 0.03 | |
| Phyla Proteobacteria richness | 46.09 | 30.15 | | 36.21 | | 18.60 | | -1.53 | | 0.126 | | 0.251 | | 0.06 | |
| Class Gammaproteobacteria richness | 22.86 | 12.13 | | 22.12 | | 14.60 | | -0.91 | | 0.361 | | 0.399 | | 0.06 | |
| Class Alphaproteobacteria richness | 23.06 | 21.89 | | 13.94 | | 7.49 | | -1.57 | | 0.116 | | 0.251 | | 0.07 | |
| Total shannon | 4.86 | 0.37 | | 4.77 | | 0.52 | | -0.90 | | 0.366 | | 0.399 | | 0.02 | |
| Phyla Proteobacteria shannon | 3.09 | 0.60 | | 2.91 | | 0.64 | | -1.81 | | 0.070 | | 0.209 | | 0.07 | |
| Class Gammaproteobacteria shannon | 2.55 | 0.64 | | 2.49 | | 0.77 | | -1.23 | | 0.219 | | 0.291 | | 0.07 | |
| Class Alphaproteobacteria shannon | 2.40 | 0.60 | | 2.01 | | 0.48 | | -2.33 | | **0.020** | | 0.118 | | 0.13 | |
| Total simpson | 0.99 | 0.00 | | 0.99 | | 0.02 | | -0.08 | | 0.937 | | 0.937 | | 0.03 | |
| Phyla Proteobacteria simpson | 0.93 | 0.05 | | 0.91 | | 0.07 | | -2.07 | | **0.039** | | 0.154 | | 0.08 | |
| Class Gammaproteobacteria simpson | 0.88 | 0.09 | | 0.86 | | 0.11 | | -1.42 | | 0.155 | | 0.266 | | 0.06 | |
| Class Alphaproteobacteria simpson | 0.86 | 0.07 | | 0.79 | | 0.12 | | -2.89 | | **0.004** | | **0.047** | | 0.15 | |

## **Table S8. Linear mixed model (LMM) results for correlation of yard plant richness with skin bacterial richness and diversity indices on the back of the hand.** Analyses were done with A) all study subjects, and B) rewilding group study subjects, and C) control study subjects at baseline and 90-days post-intervention. LMM statistics are reported as t value, p value, Benjamini-Hochberg adjusted p value and R squared (R^2^).

| A) All study subjects | Baseline | | | | 90-days post-intervention | | | |
| --- | --- | --- | --- | --- | --- | --- | --- | --- |
|  | **t value** | **p value** | **p adjust** | **R^2^** | **t value** | **p value** | **p adjust** | **R^2^** |
| Total richness | 1.42 | 0.154 | 0.277 | 0.07 | 2.59 | **0.010** | **0.030** | 0.18 |
| Gammaproteobacteria richness | 0.10 | 0.920 | 0.920 | 0.00 | 0.51 | 0.611 | 0.611 | 0.01 |
| Alphaproteobacteria richness | 1.94 | 0.053 | 0.119 | 0.20 | 0.86 | 0.392 | 0.588 | 0.02 |
| Total Shannon | 0.82 | 0.410 | 0.553 | 0.03 | 3.11 | **0.002** | **0.018** | 0.24 |
| Gammaproteobacteria Shannon | 0.40 | 0.686 | 0.772 | 0.00 | 0.73 | 0.465 | 0.598 | 0.02 |
| Alphaproteobacteria Shannon | 2.69 | **0.007** | **0.032** | 0.24 | 1.36 | 0.174 | 0.313 | 0.06 |
| Total Simpson | 2.21 | **0.027** | 0.081 | 0.17 | 2.67 | **0.008** | **0.030** | 0.22 |
| Gammaproteobacteria Simpson | 0.79 | 0.430 | 0.553 | 0.04 | 0.51 | 0.610 | 0.611 | 0.01 |
| Alphaproteobacteria Simpson | 2.71 | **0.007** | **0.032** | 0.20 | 2.00 | **0.046** | 0.104 | 0.12 |
| B) Rewilding | **Baseline** | | | | **90-days post-intervention** | | | |
|  | **t value** | **p value** | **p adjust** | **R^2^** | **t value** | **p value** | **p adjust** | **R^2^** |
| Total richness | 1.84 | 0.081 | 0.223 | 0.15 | 3.10 | **0.006** | **0.027** | 0.34 |
| Gammaproteobacteria richness | 0.94 | 0.361 | 0.370 | 0.04 | 0.27 | 0.789 | 0.789 | 0.00 |
| Alphaproteobacteria richness | 0.92 | 0.370 | 0.370 | 0.04 | 0.77 | 0.450 | 0.537 | 0.03 |
| Total Shannon | 1.28 | 0.217 | 0.279 | 0.08 | 3.32 | **0.004** | **0.027** | 0.37 |
| Gammaproteobacteria Shannon | 1.35 | 0.193 | 0.279 | 0.09 | 0.73 | 0.477 | 0.537 | 0.03 |
| Alphaproteobacteria Shannon | 1.52 | 0.146 | 0.263 | 0.11 | 1.09 | 0.291 | 0.437 | 0.06 |
| Total Simpson | 1.97 | 0.064 | 0.223 | 0.17 | 2.62 | **0.017** | **0.051** | 0.26 |
| Gammaproteobacteria Simpson | 1.93 | 0.069 | 0.223 | 0.16 | 1.33 | 0.200 | 0.360 | 0.08 |
| Alphaproteobacteria Simpson | 1.74 | 0.099 | 0.223 | 0.14 | 1.41 | 0.175 | 0.360 | 0.09 |
| C) Control | **Baseline** | | | | **90-days post-intervention** | | | |
|  | **t value** | **p value** | **p adjust** | **R^2^** | **t value** | **p value** | **p adjust** | **R^2^** |
| Total richness | -0.24 | 0.813 | 0.838 | 0.00 | 0.09 | 0.933 | 0.992 | 0.00 |
| Gammaproteobacteria richness | -1.19 | 0.255 | 0.540 | 0.10 | -0.51 | 0.620 | 0.872 | 0.03 |
| Alphaproteobacteria richness | 1.18 | 0.257 | 0.540 | 0.10 | 0.48 | 0.639 | 0.872 | 0.03 |
| Total Shannon | -0.21 | 0.838 | 0.838 | 0.00 | 0.43 | 0.678 | 0.872 | 0.02 |
| Gammaproteobacteria Shannon | -1.08 | 0.300 | 0.540 | 0.08 | -0.70 | 0.500 | 0.872 | 0.05 |
| Alphaproteobacteria Shannon | 1.77 | 0.099 | 0.446 | 0.19 | 0.75 | 0.474 | 0.872 | 0.06 |
| Total Simpson | 0.36 | 0.725 | 0.838 | 0.01 | -0.01 | 0.992 | 0.992 | 0.00 |
| Gammaproteobacteria Simpson | -0.93 | 0.367 | 0.551 | 0.06 | -0.91 | 0.386 | 0.872 | 0.08 |
| Alphaproteobacteria Simpson | 2.04 | 0.062 | 0.446 | 0.24 | 1.32 | 0.219 | 0.872 | 0.16 |

## **Table S9.** ANCOM-BC2 results for the Treatment x Time interaction for (A) oral microbiota, (B) oral functional gene pathways, (C) skin bacterial genera on the back of the hand and (D) on forearm.

Effect estimates are reported as log fold change for Tretmant x Time interaction (LFC), along with standard error (SE), Wald test statistic, p-value, and Benjamini–Hochberg adjusted p-value (q-value). The sample-size screening criterion (passed ss) and the robustness indicator (diff robust) are also reported.

| A) Oral microbial taxa | LFC | SE | Wald | p value | q value | passed ss | diff robust |
| --- | --- | --- | --- | --- | --- | --- | --- |
| *Lautropia mirabilis* | 5.36 | 2.12 | 2.53 | **0.015** | **0.049** | TRUE | FALSE |
| *Anaerostipes hadrus* | 1.92 | 0.59 | 2.58 | **0.016** | 0.052 | TRUE | FALSE |
| *Chryseobacterium indologenes* | -1.89 | 0.75 | -2.53 | **0.016** | 0.054 | TRUE | FALSE |
| *Kaistella flava* | -1.67 | 0.64 | -2.62 | **0.017** | 0.054 | TRUE | FALSE |
| *Alistipes indistinctus* | 1.45 | 0.57 | 2.55 | **0.017** | 0.055 | TRUE | FALSE |
| *Sodaliphilus pleomorphus* | 1.53 | 0.77 | 2.50 | **0.017** | 0.055 | TRUE | FALSE |
| *Bacillus sp. Y1* | 1.16 | 0.45 | 2.57 | **0.018** | 0.057 | TRUE | FALSE |
| B) Oral functional gene pathways | **LFC** | **SE** | **Wald** | **p value** | **q value** | **passed ss** | **diff robust** |
| PWY 6612 superpathway of tetrahydrofolate biosynthesis unclassified | -4.16 | 0.61 | -6.87 | **0.0001** | **0.016** | TRUE | FALSE |
| FOLSYN PWY superpathway of tetrahydrofolate biosynthesis and salvage unclassified | -3.79 | 0.58 | -6.51 | **0.0003** | **0.021** | TRUE | FALSE |
| P42 PWY incomplete reductive TCA cycle unclassified | -2.23 | 0.32 | -7.06 | **0.001** | **0.037** | TRUE | FALSE |
| HISDEG PWY L histidine degradation I | -2.51 | 0.69 | -3.62 | **0.002** | **0.047** | TRUE | FALSE |
| PWY 5188 tetrapyrrole biosynthesis I from glutamate unclassified | -1.65 | 0.51 | -3.27 | **0.003** | 0.076 | TRUE | FALSE |
| PWY0 1297 superpathway of purine deoxyribonucleosides degradation | -2.30 | 0.81 | -2.85 | **0.006** | 0.090 | TRUE | FALSE |
| PWY 7238 sucrose biosynthesis II g Haemophilus.s Haemophilus parainfluenzae | 2.00 | 0.32 | 6.35 | **0.008** | 0.090 | TRUE | FALSE |
| C) Skin bacterial genera, hand | **LFC** | **SE** | **Wald** | **p value** | **q value** | **passed ss** | **diff robust** |
| *Rothia* | -2.02 | 0.75 | -2.67 | **0.011** | **0.039** | TRUE | FALSE |
| *Rhodococcus* | 1.77 | 0.60 | 2.96 | **0.011** | **0.039** | TRUE | FALSE |
| *Clostridium sensu stricto 13* | -1.60 | 0.50 | -3.21 | **0.012** | **0.043** | TRUE | FALSE |
| *Marmoricola* | 3.90 | 0.46 | 8.54 | **0.013** | **0.044** | TRUE | FALSE |
| *Peptoniphilus* | -2.30 | 0.90 | -2.56 | **0.014** | **0.044** | TRUE | FALSE |
| *Oribacterium* | 1.90 | 0.49 | 3.88 | **0.018** | 0.056 | TRUE | FALSE |
| *Bosea* | -1.62 | 0.50 | -3.23 | **0.023** | 0.072 | TRUE | FALSE |
| D) Skin bacterial genera, forearm | **LFC** | **SE** | **Wald** | **p value** | **q value** | **passed ss** | **diff robust** |
| *Sphingobacterium* | 2.51 | 0.84 | 2.99 | **0.010** | **0.042** | TRUE | FALSE |
| *Peredibacter* | -5.28 | 0.55 | -9.68 | **0.011** | **0.044** | TRUE | FALSE |
| *Lawsonella* | 2.21 | 0.83 | 2.65 | **0.011** | **0.044** | TRUE | FALSE |
| *Dyadobacter* | -2.08 | 0.63 | -3.28 | **0.014** | 0.052 | TRUE | FALSE |
| *Negativicoccus* | -2.90 | 0.58 | -5.03 | **0.015** | 0.056 | TRUE | FALSE |
| *Solobacterium* | 2.77 | 0.57 | 4.88 | **0.016** | 0.059 | TRUE | FALSE |
| *Romboutsia* | 3.88 | 0.56 | 6.96 | **0.020** | 0.070 | TRUE | FALSE |

## Table S10. Correlation between salivary cytokines (IL-6 and IL-10) and yard characteristics, and berry picking within A) rewilding and B) control treatment, and with C) all study subjects in the GLM model.

Data is presented as mean ± standard deviation (sd). GLM statistics are reported as t value, p value and Benjamini-Hochberg adjusted p value (padj).

| A) Rewilding | Baseline | | | | | | Fall (day 90) | | | | | |
| --- | --- | --- | --- | --- | --- | --- | --- | --- | --- | --- | --- | --- |
| IL-6 | mean | sd | t value | p value | padj | R^2^ | mean | sd | t value | p value | padj | R^2^ |
| Berry and fruit picking | 1.7 | 0.9 | -0.9 | 0.384 | 0.750 | 0.05 | 1.6 | 1.1 | 3.2 | **0.006** | **0.017** | 0.52 |
| Fruit trees% | 6.3 | 10.7 | 4.5 | **<0.0001** | **0.012** | 0.88 | 4.6 | 7.4 | 0.5 | 0.600 | 0.997 | 0.008 |
| Tree canopy% | 16.9 | 17.7 | 2.7 | **0.015** | 0.120 | 0.37 | 15.2 | 16.4 | 1.7 | 0.110 | 0.524 | 0.10 |
| Plant richness | 82.6 | 30.9 | 3.2 | **0.005** | 0.056 | 0.37 | 97.6 | 28.6 | 1.1 | 0.293 | 0.624 | 0.07 |
| IL-10 | mean | sd | t value | p value | padj | R^2^ | mean | sd | t value | p value | padj | R^2^ |
| Berry and fruit picking | 1.7 | 0.9 | -1.2 | 0.228 | 0.669 | 0.09 | 1.6 | 1.1 | 3.6 | **0.002** | **0.016** | 0.32 |
| Fruit trees% | 6.3 | 10.7 | 2.4 | **0.029** | 0.399 | 0.47 | 4.6 | 7.4 | 0.5 | 0.646 | 0.997 | 0.01 |
| Tree canopy% | 16.9 | 17.7 | 2.1 | **0.047** | 0.448 | 0.26 | 15.2 | 16.4 | 1.8 | 0.083 | 0.395 | 0.09 |
| Plant richness | 82.6 | 30.9 | 2.3 | **0.034** | 0.410 | 0.22 | 97.6 | 28.6 | 0.7 | 0.492 | 0.997 | 0.02 |
| B) Control | **Baseline** | | | | | | **Fall (day 90)** | | | | | |
| IL-6 | mean | sd | t value | p value | padj | R^2^ | mean | sd | t value | p value | padj | R^2^ |
| Berry and fruit picking | 2.3 | 1.8 | 1.8 | 0.105 | 0.706 | 0.20 | 2.0 | 1.6 | 1.3 | 0.241 | 0.812 | 0.14 |
| Fruit trees% | 3.7 | 5.9 | -0.9 | 0.377 | 0.706 | 0.14 | 3.7 | 5.9 | -0.6 | 0.548 | 0.899 | 0.02 |
| Tree canopy% | 12.0 | 10.4 | -1.1 | 0.278 | 0.706 | 0.14 | 12.0 | 10.4 | 0.0 | 0.992 | 0.998 | <0.001 |
| Plant richness | 89.4 | 24.3 | 1.7 | 0.126 | 0.706 | 0.16 | 89.4 | 24.3 | 1.9 | 0.087 | 0.395 | 0.50 |
| IL-10 | mean | sd | t value | p value | padj | R^2^ | mean | sd | t value | p value | padj | R^2^ |
| Berry and fruit picking | 2.3 | 1.8 | 0.7 | 0.481 | 0.812 | 0.05 | 2.0 | 1.6 | -0.2 | 0.831 | 0.997 | 0.002 |
| Fruit trees | 3.7 | 5.9 | 0.4 | 0.710 | 0.914 | 0.02 | 3.7 | 5.9 | -0.7 | 0.513 | 0.998 | 0.03 |
| Tree canopy | 12.0 | 10.4 | -0.7 | 0.519 | 0.812 | 0.07 | 12.0 | 10.4 | -1.1 | 0.313 | 0.998 | 0.04 |
| Plant richness | 89.4 | 24.3 | 0.4 | 0.668 | 0.743 | 0.02 | 89.4 | 24.3 | 0.8 | 0.430 | 0.812 | 0.06 |
| C) All study subjects | **Baseline** | | | | | | **Fall (day 90)** | | | | | |
| IL-6 | mean | sd | t value | p value | padj | R^2^ | mean | sd | t value | p value | padj | R^2^ |
| Berry and fruit picking | 1.9 | 1.4 | -0.5 | 0.625 | 0.883 | 0.01 | 1.8 | 1.2 | 3.1 | **0.005** | 0.057 | 0.45 |
| Fruit trees% | 5.3 | 9.2 | 5.5 | **<0.0001** | **<0.0001** | 0.82 | 4.2 | 6.8 | -0.1 | 0.921 | 0.987 | 0.002 |
| Tree canopy% | 15.1 | 15.4 | 3.4 | **0.002** | **0.021** | 0.32 | 13.9 | 14.3 | 1.1 | 0.298 | 0.868 | 0.03 |
| Plant richness | 85.1 | 28.4 | 3.8 | **0.001** | **0.002** | 0.29 | 95.9 | 27.8 | 2.0 | 0.055 | 0.128 | 0.12 |
| IL-10 | mean | sd | t value | p value | padj | R^2^ | mean | sd | t value | p value | padj | R^2^ |
| Berry and fruit picking | 1.9 | 1.4 | -0.2 | 0.840 | 0.913 | 0.001 | 1.8 | 1.3 | 0.8 | 0.406 | 0.987 | 0.07 |
| Fruit trees% | 5.3 | 9.2 | 2.6 | **0.015** | 0.249 | 0.33 | 4.2 | 6.8 | -0.2 | 0.827 | 0.987 | 0.002 |
| Tree canopy% | 15.1 | 15.4 | 1.8 | 0.074 | 0.578 | 0.15 | 13.9 | 14.3 | 0.2 | 0.839 | 0.987 | 0.002 |
| Plant richness | 85.1 | 28.4 | 2.2 | **0.032** | 0.249 | 0.14 | 95.9 | 27.8 | 1.0 | 0.333 | 0.987 | 0.02 |

## **Table S11.** Garden plants were chosen based on their bud microbial richness and Shannon-Wiener and Simpson diversity indexes.

Data is presented as mean ± standard deviation (sd) (n = 3).

|  | Richness | | Shannon index | | Simpson index | |
| --- | --- | --- | --- | --- | --- | --- |
|  | **Mean** | **sd** | **Mean** | **sd** | **Mean** | **sd** |
| Amelanchier laevis | 427.67 | 370.61 | 3.56 | 0.86 | 0.10 | 0.07 |
| Chaenomeles japonica Venus | 673.67 | 90.26 | 4.07 | 0.29 | 0.04 | 0.01 |
| Crataegus x media Pauls Scarlet | 620.33 | 258.70 | 4.33 | 0.80 | 0.06 | 0.05 |
| Cytisus decumbens Niilo | 125.67 | 20.82 | 3.74 | 0.18 | 0.05 | 0.01 |
| Forsythia mandshurica | 340.33 | 137.26 | 4.09 | 0.38 | 0.07 | 0.05 |
| Kolkwitzia amabilis | 452.33 | 106.71 | 4.31 | 0.15 | 0.03 | 0.01 |
| Lonicera caerulea | 106.33 | 50.29 | 3.13 | 0.41 | 0.08 | 0.03 |
| Malus Lobo | 559.33 | 155.16 | 3.55 | 0.53 | 0.10 | 0.07 |
| Malus Sandra | 663.00 | 59.67 | 4.11 | 0.16 | 0.05 | 0.01 |
| Malus toringo var.sargentii | 484.00 | 97.20 | 3.90 | 0.19 | 0.05 | 0.01 |
| Prunus cerasus Rauhalan morelli | 727.67 | 100.45 | 3.81 | 0.40 | 0.07 | 0.03 |
| Ribes Rubrum, Piikkion Helmi | 255.00 | 19.30 | 3.87 | 0.05 | 0.05 | 0.00 |
| Ribes Rubrum, Punahilkka | 215.33 | 7.41 | 4.04 | 0.07 | 0.04 | 0.01 |
| Ribes uva crispa Hinnonmaen keltainen | 217.33 | 26.13 | 3.87 | 0.16 | 0.04 | 0.01 |
| Ribes uva crispa Lepaan punainen | 429.67 | 132.71 | 4.12 | 0.19 | 0.03 | 0.00 |
| Rosa Gallica Ryhma Olkkala | 192.67 | 86.68 | 2.96 | 0.10 | 0.11 | 0.02 |
| Rosa majalis Tornedal | 119.00 | 15.51 | 3.35 | 0.12 | 0.08 | 0.01 |
| Rubus caesius | 273.50 | 35.50 | 2.60 | 0.07 | 0.17 | 0.04 |
| Salix glauca var. callicarpaea Haltia | 348.33 | 163.04 | 3.09 | 0.74 | 0.16 | 0.09 |
| Sorbus x thuringiaca | 439.00 | 49.00 | 3.81 | 0.21 | 0.09 | 0.02 |
| Spiraea alba Allikko | 629.33 | 134.11 | 3.73 | 0.19 | 0.06 | 0.02 |
| Spiraea japonica Lilly | 445.67 | 50.89 | 4.14 | 0.09 | 0.03 | 0.00 |
| Spiraea japonica Little Princess | 490.00 | 149.53 | 4.00 | 0.19 | 0.04 | 0.01 |
| Spiraea media | 509.33 | 221.07 | 4.19 | 0.09 | 0.04 | 0.01 |
| Spiraea x watsoniana Kruunu | 653.33 | 28.55 | 3.88 | 0.04 | 0.05 | 0.01 |
| Vaccinium corymbosum Arto | 66.67 | 27.82 | 2.42 | 0.11 | 0.20 | 0.02 |
| Weigela middendorffiana Jouni | 549.00 | 92.40 | 3.92 | 0.36 | 0.05 | 0.02 |


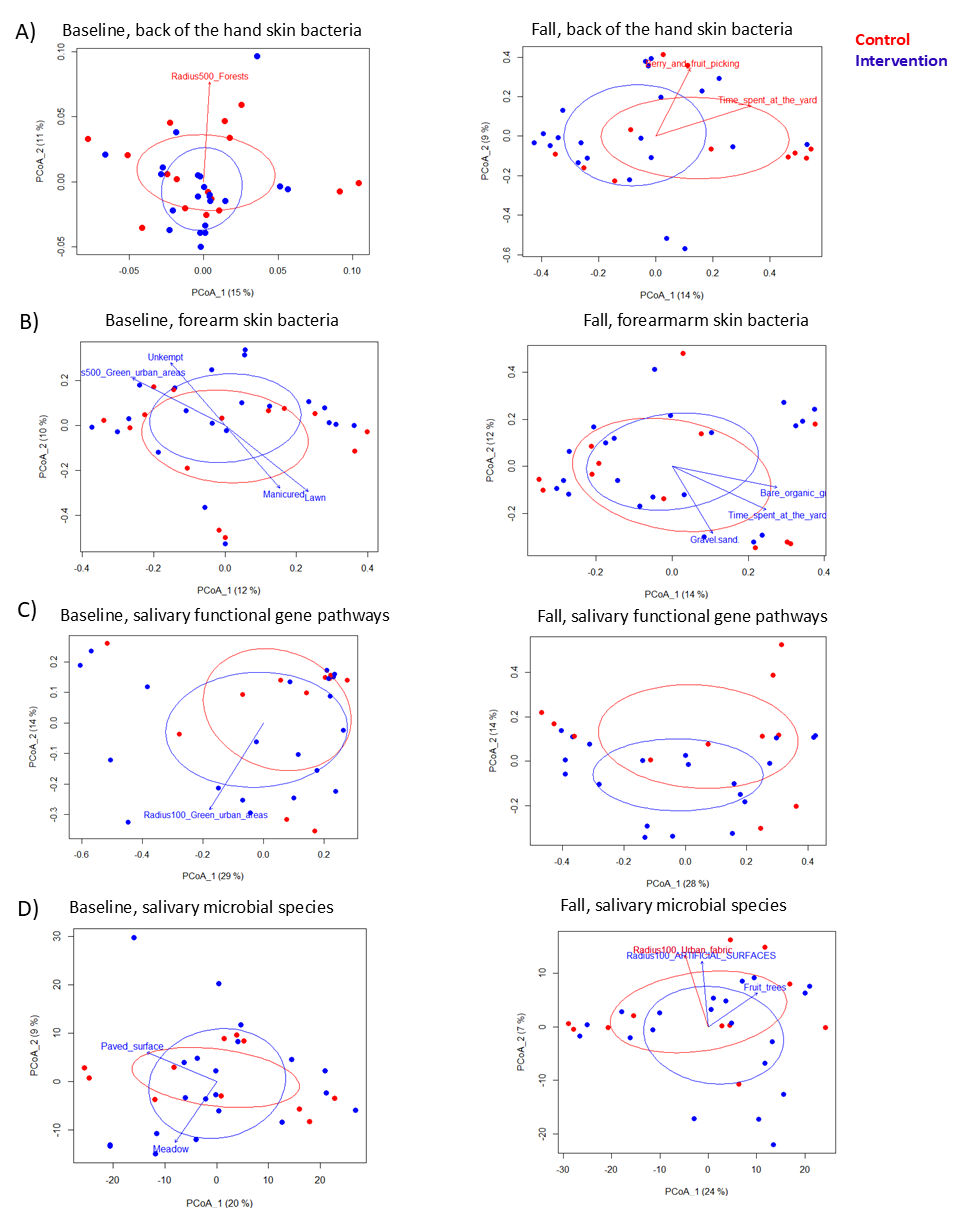


## Figure S1. Principal coordinated analysis (PCoA) for microbial beta diversities between rewilding and control group.

PCoA plots are calculated with Bray-Curtis distance at ASV level (abundance data i.e. weighted data) for A) skin bacteria on the back of the hand and B) on forearm, and for C) salivary functional gene pathways and C) salivary microbial species at baseline and in fall (day 90). The correlation between environmental variables and microbial beta diversity is tested using function envfit in vegan package. Ellipses represent one standard deviation around the centroid of each treatment group in PCoA space. Blue arrow *p* < 0.05 and red arrow adjusted padj < 0.05. Significance is based on permutation tests.


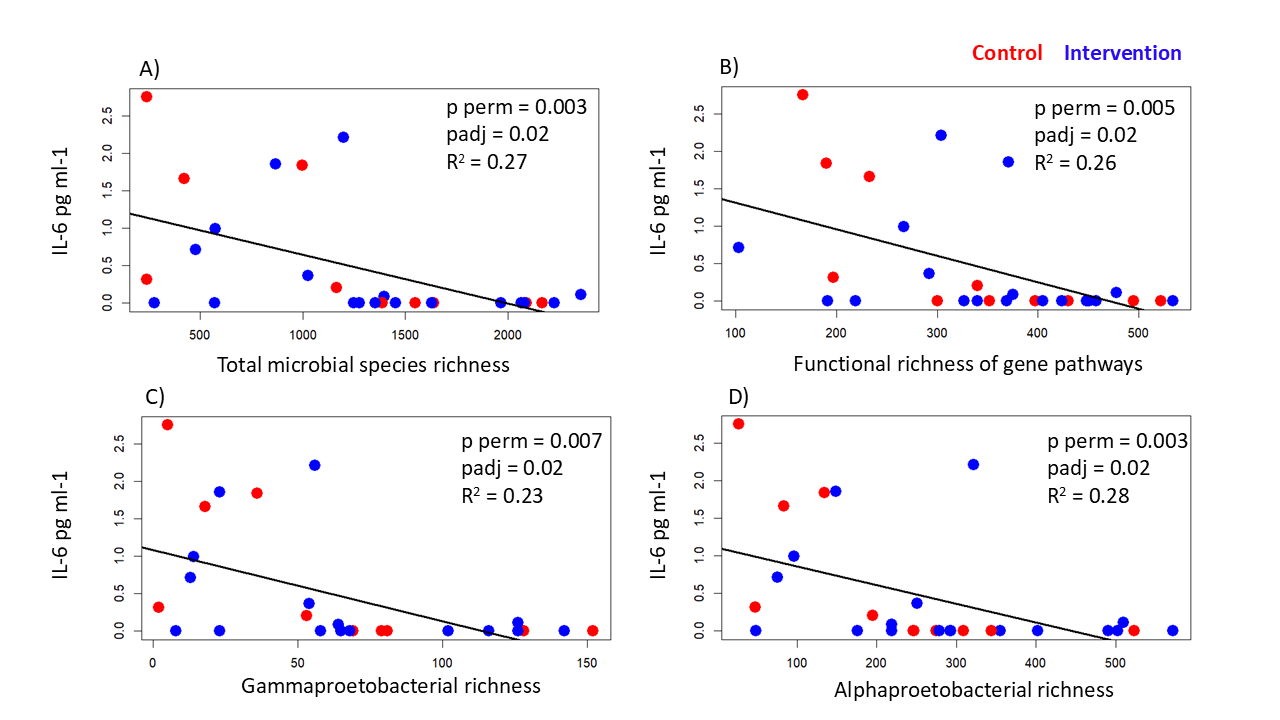


## Figure S2. Association between salivary IL-6 and A) total salivary microbial richness, B) richness of functional gene pathways in saliva, and C) salivary gammaproteobacterial and D) alphaproteobacterial richness. Associations were evaluated using linear mixed-effects models, with subject nested within city included as a random effect. Statistical significance was assessed using permutation testing (5,000 permutations) to derive empirical p-values. The figure displays permutation-based p-values (p perm), Benjamini–Hochberg false discovery rate–adjusted p-values (padj), and model R² values.


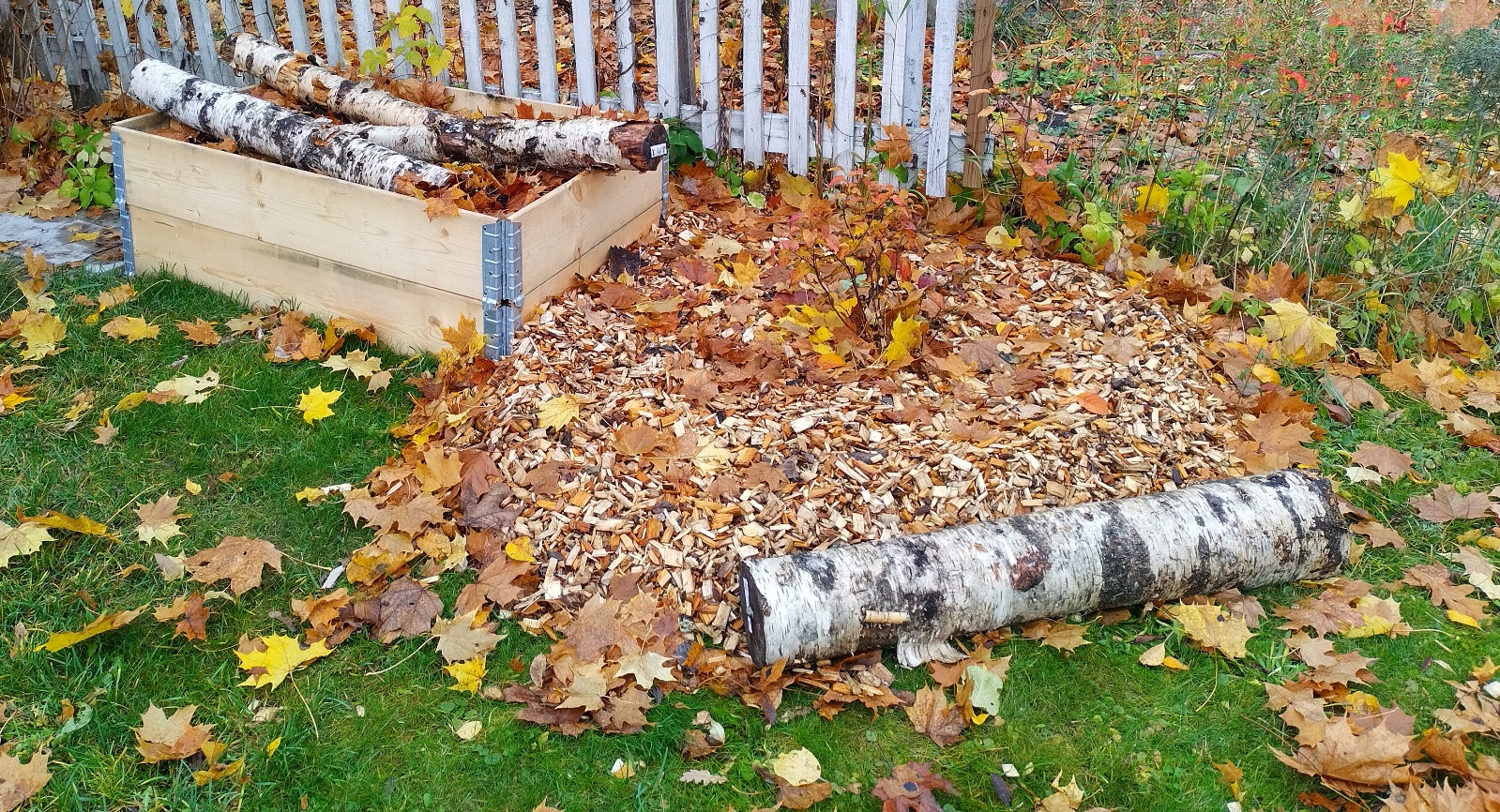


## Figure S3. Cultivation box installed in a rewilding yard. The box contained three 1-m logs of silver birch (*Betula pendula*) inoculated two years prior to the intervention with lion’s mane (*Hericium erinaceus*) and shiitake (*Lentinula edodes*) fungi. Wood chips composed of birch, aspen and oak were also transferred to the rewilded yards. Participants in the rewilding group were instructed to leave autumn leaves on site to decompose naturally.

# References

1 Harris PA, Taylor R, Minor BL, *et al.* The REDCap consortium: Building an international community of software platform partners. *Journal of Biomedical Informatics* 2019; **95**: 103208.

2 Harris PA, Taylor R, Thielke R, Payne J, Gonzalez N, Conde JG. Research electronic data capture (REDCap)—A metadata-driven methodology and workflow process for providing translational research informatics support. *Journal of Biomedical Informatics* 2009; **42**: 377–81.

3 Hanski I, von Hertzen L, Fyhrquist N, *et al.* Environmental biodiversity, human microbiota, and allergy are interrelated. *Proc Natl Acad Sci USA* 2012; **109**: 8334–9.

4 Roslund MI, Puhakka R, Grönroos M, *et al.* Biodiversity intervention enhances immune regulation and health-associated commensal microbiota among daycare children. *Sci Adv* 2020; **6**: eaba2578.

5 Roslund MI, Puhakka R, Nurminen N, *et al.* Long-term biodiversity intervention shapes health-associated commensal microbiota among urban day-care children. *Environment International* 2021; **157**: 106811.

6 Roslund MI, Parajuli A, Hui N, *et al.* A Placebo-controlled double-blinded test of the biodiversity hypothesis of immune-mediated diseases: Environmental microbial diversity elicits changes in cytokines and increase in T regulatory cells in young children. *Ecotoxicology and Environmental Safety* 2022; **242**: 113900.

7 Roslund MI, Puhakka R, Grönroos M, *et al.* Biodiversity intervention enhances immune regulation and health-associated commensal microbiota among daycare children. *Science advances* 2020; **6**. DOI:10.1126/sciadv.aba2578.

8 Hayes RJ, Bennett S. Simple sample size calculation for cluster-randomized trials. *International Journal of Epidemiology* 1999; **28**: 319–26.
